# Postbiotic-Mediated Green Synthesis of Silver Nanoparticles: Revealing Potent Antimicrobial, Anti-Biofilm, Antioxidant, and Anticancer Properties using *Paenibacillus xylanexedens*

**DOI:** 10.1007/s12602-025-10905-8

**Published:** 2026-02-04

**Authors:** Sema Yiyit Doğan, Seçil Kaya, Ebru Kondolot Solak

**Affiliations:** 1 Vocational School of Technical Sciences, Gazi University, Ankara, 06374 Turkey; 2 Vocational School of Technical Sciences, Department of Chemistry and Chemical Processing Technologies, Gazi University, Ankara, 06374 Turkey; 3 Vocational School of Technical Sciences, Department of Material and Material Processing Technologies, Gazi University, Ankara, 06374 Turkey

**Keywords:** Green synthesis, Postbiotic, Nanoparticles, Antimicrobial, Antioxidant activity, Cytotoxicity

## Abstract

This study aimed to synthesize and characterize silver nanoparticles (AgNPs) using the postbiotic of *Paenibacillus xylanexedens* YSM1 and to evaluate their antimicrobial, antioxidant, and anticancer activities. The extracellular postbiotic-mediated synthesis provides a simple, non-toxic, and sustainable route that eliminates the need for chemical reducing agents while enhancing nanoparticle biocompatibility. Optimization studies using UV–visible spectroscopy identified the optimal synthesis conditions as 3 mM AgNO₃ concentration, a 1:5 AgNO₃-to-postbiotic ratio, 360 min reaction time, and 60 °C incubation temperature. The distinct color change from pale yellow to dark brown confirmed AgNP formation, with a characteristic surface plasmon resonance (SPR) peak at 435 nm. FT-IR analysis revealed hydroxyl, amide, and carbonyl groups, indicating the involvement of postbiotic metabolites as natural reducing and capping agents. The biosynthesized AgNPs exhibited notable antimicrobial activity against*Escherichia coli*,*Staphylococcus aureus*, and *Candida albicans*, with MIC values of 62.5, 125, and 250 µg/mL, respectively. In addition, strong antibiofilm activity was observed against bacterial biofilms, achieving ≥75% inhibition for *E. coli* and *S. aureus* at concentrations≥62.5 µg/mL, while *C. albicans* biofilms required higher concentrations to reach comparable inhibition. The antibiofilm-effective concentrations showed close agreement with planktonic MIC values, indicating a coherent antimicrobial–antibiofilm relationship. DPPH assays demonstrated concentration-dependent radical scavenging activity, reaching nearly 100% inhibition at 500 µg/mL, confirming the antioxidant potential of postbiotic nanoparticles. Cytotoxicity studies performed on HT-29 colorectal adenocarcinoma and MRC-5 normal fibroblast cell lines revealed selective toxicity toward cancer cells (IC₅₀ ≈ 125 µg/mL) while maintaining more than 92% viability in normal cells.

## Introduction

 Biologically synthesised nanoparticles are attracting increasing attention in biomedical research due to their versatility, environmentally friendly production methods, natural biocompatibility, and multifunctional bioactivities. In recent years, nanotechnology-focused approaches have shown significant promise in biomedical contexts, particularly through the development of antimicrobial, antioxidant, and anticancer nanomaterials capable of overcoming the limitations of conventional therapeutics [1, 2]. Beyond biomedical applications, biogenic nanoparticles have also demonstrated notable potential in non-medical sectors. Recent studies report their ability to enhance food packaging performance by inhibiting spoilage-causing microorganisms and extending shelf life through controlled antimicrobial release [3]. Additionally, when used as agricultural nanobiostimulants or nano-fertilisers, biogenic nanoparticles have been shown to improve plant growth, nutrient uptake, and stress tolerance, highlighting their relevance in sustainable agriculture and environmental biotechnology [4].

Silver nanoparticles (AgNPs) are being explored as potential alternative materials for treating various diseases such as cancer, wound healing, and infectious diseases due to their unique chemical and physical properties [5–9]. These properties of AgNPs are attributed to their small size, large specific surface area, high surface atom ratio, and broad-spectrum antimicrobial activity [9, 10]. Various physical, chemical, and biological synthesis methods have been reported to prepare AgNPs [9–12]. However, physical and chemical methods have several disadvantages, including high costs, high energy requirements, the use of toxic chemicals, and the production of hazardous waste [13, 14]. It is therefore evident that there is an increasing need for environmentally friendly methods of synthesizing nanoparticles. Biogenic AgNPs are synthesized through plant extracts [15], microorganisms [16], algae [17], fungi [18], secondary metabolites [12–19], and other biological products [20, 16]. The distinguishing features of this biological method include its reduced toxicity, energy efficiency, sustainability, reduced waste generation, enhanced nanoparticle stability, and alignment with green nanotechnology principles [14–21].

Microbial synthesis of AgNPs in bacteria is divided into intracellular and extracellular, depending on where the nanoparticles are produced [22, 23]. The most accepted intracellular synthesis mechanism is nitrate is converted to nitrite by bacterial cell walls and bacterial cell wall enzymes such as nitrate reductase. While alpha-nicotinamide adenine dinucleotide phosphate reduced form (NADPH)-dependent nitrate reductase converts nitrate to nitrite, it transfers an electron to the silver ion (Ag⁺), and thus the Ag⁺ is reduced to the neutral silver atom (Ag⁰) [24–27]. Studies have also shown that AgNPs accumulate due to electrostatic interactions in bacteria’s periplasm, cytoplasm, and cell wall components [25–29]. The extracellular synthesis mechanism is carried out in bacteria via the ability to secrete various bioactive compounds, including enzymes, proteins, hormones, ions, polysaccharides, pigments, and secondary metabolites. Numerous bacterial strains such as *Bacillus sp.* [29, 30], *Pseudomonas sp.* [31, 32], and *Acinetobacter sp.* [33] have been shown to synthesize silver nanoparticles via their secondary metabolites. One genus of particular interest is *Paenibacillus*, formerly classified under *Bacillus* but now recognized as a separate genus [34, 35].

*Paenibacillus* species are characterized by their gram-positive, facultative anaerobic, endospore-forming nature [34]– [35]. These organisms are distinguished by their capacity to produce exopolysaccharides and extracellular enzymes of significant industrial relevance. They are employed as biocontrol agents and to enhance plant growth [34–36]. *Paenibacillus* strains have been recognized as probiotic bacteria in recent years. Certain *Paenibacillus* strains, such as *P. polymyxa*, *P. konkukensis*, and *P. xylanexedens*, exhibit antimicrobial, antioxidant, and anticarcinogenic properties [37–40] further supporting their consideration as next-generation probiotics. Previous research has shown that *P. xylanexedens* YSM1 is effective against pathogens such as *E. coli*, *S. aureus*, *Klebsiella pneumoniae*, and *Pseudomonas aeruginosa*. Additionally, dietary inclusion of this probiotic has been found to improve and regulate intestinal health in animals [41, 42]. Despite the vast applications of *P. xylanexedens* species in agriculture and husbandry and their antimicrobial and antifungal properties, no studies have been found regarding their nanoparticle synthesis capabilities. The current literature on *Paenibacillus*-mediated AgNPs is limited in scope and quantity. As illustrated in Table 1, a summary of the studies conducted on *Paenibacillus*-mediated AgNPs research is presented. As a result of the examinations, there are no studies in the literature on the synthesis of silver nanoparticles via *P. xylanexedens*.

**Table 1:**
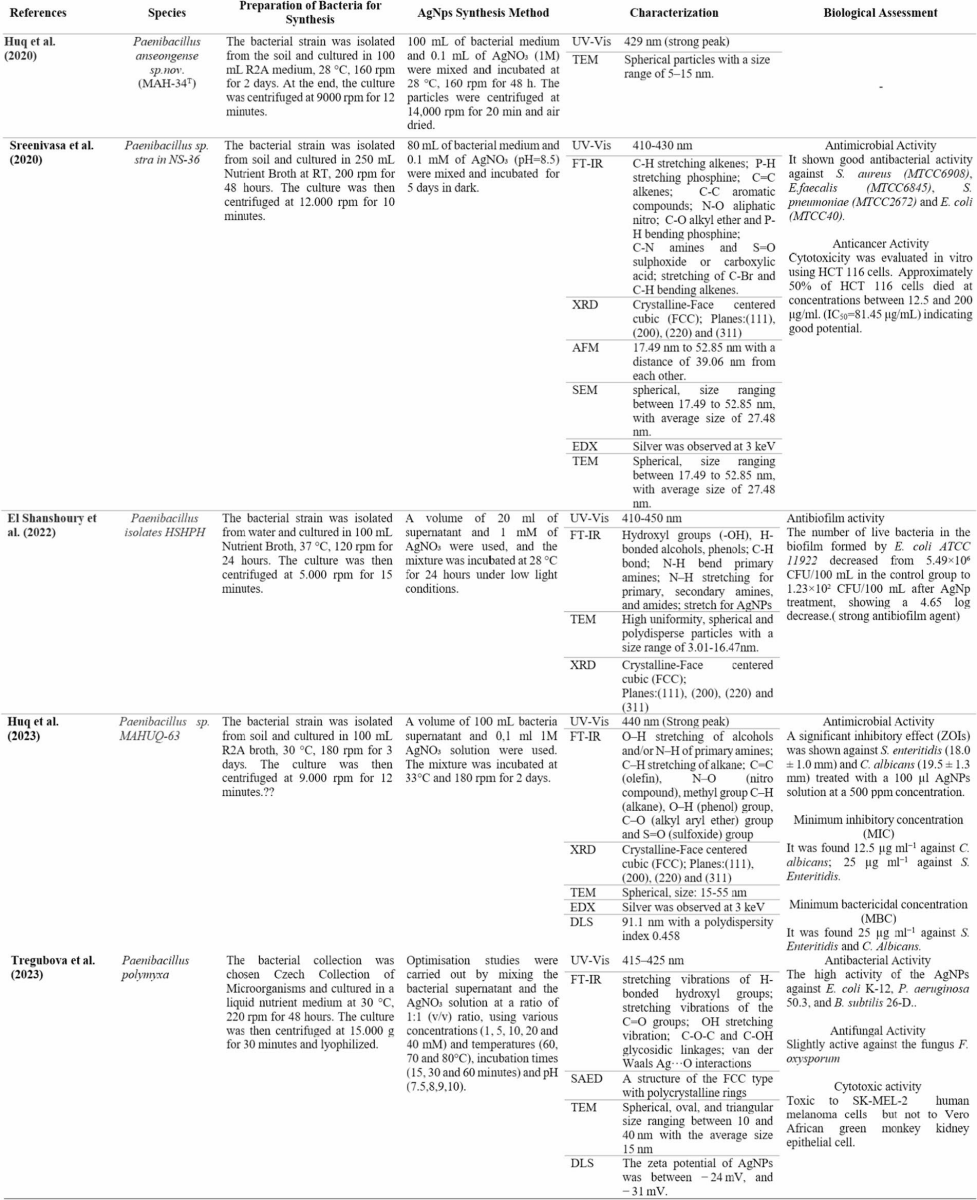
*Paenibacillus-*mediated AgNps synthesis studies in the literature

**Fig. 1:**
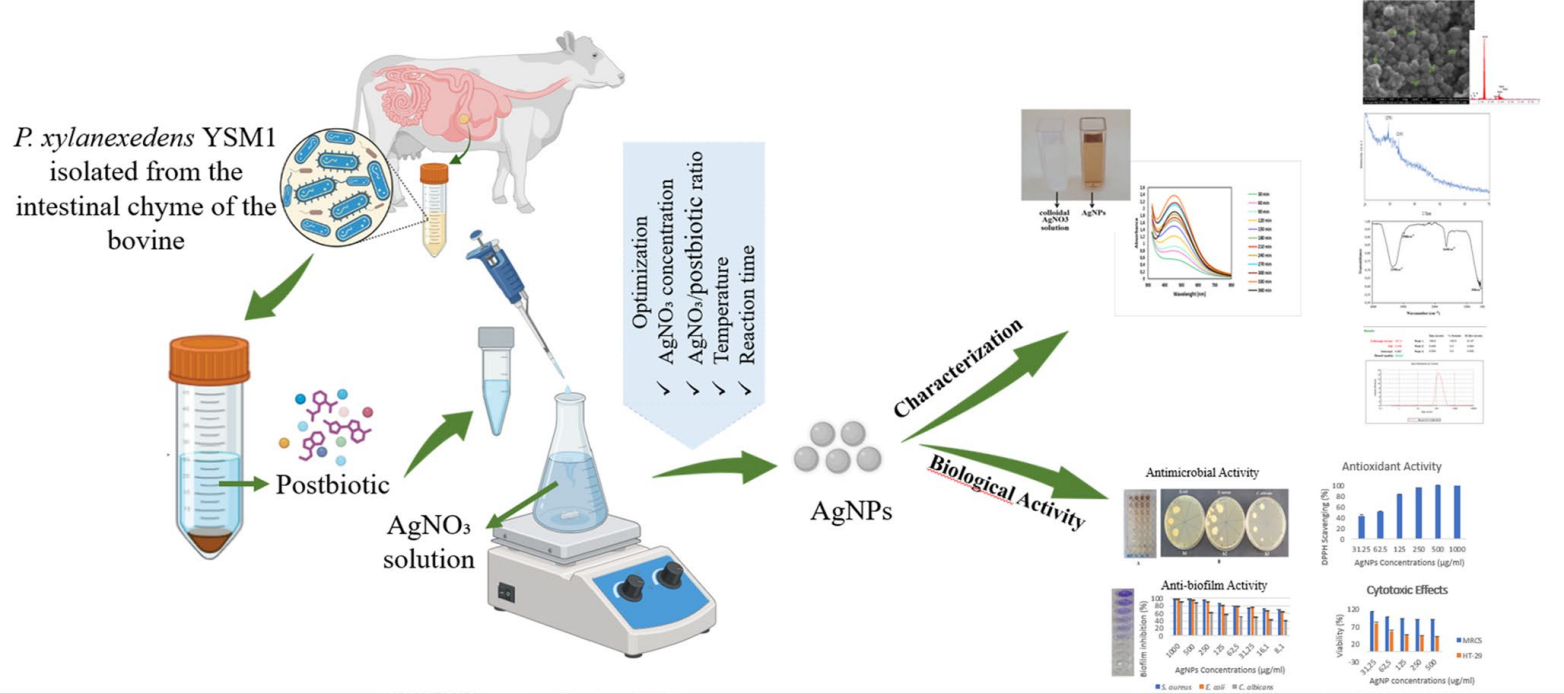
Schematic overview of the postbiotic-mediated green synthesis of silver nanoparticles (AgNPs) using *P. xylanexedens* YSM1 and the evaluation of their antimicrobial, anti-biofilm, antioxidant, and anticancer properties

Despite the documented probiotic properties of *P. xylanexedens*, no studies to date have investigated its capacity for nanoparticle synthesis. Given its proven ability to secrete bioactive compounds with antimicrobial and immunomodulatory effects, we hypothesized that *P. xylanexedens* YSM1 could serve as a promising biological source for the green synthesis of AgNPs. To the best of our knowledge, this is the first study reporting the biosynthesis of AgNPs using *P. xylanexedens* YSM1 postbiotics. Here, we optimized the synthesis conditions, performed extensive physicochemical characterization (UV–Vis, FTIR, SEM, EDX, XRD), and evaluated the biological properties of the resulting AgNPs, including antimicrobial, antioxidant, antibiofilm, and anticancer activities (Fig. 1). Our findings expand the current knowledge of *Paenibacillus*-mediated nanomaterials and suggest that *P. xylanexedens*-derived AgNPs hold significant potential for biomedical applications.

## Materials and Methods

### Materials

The *P. xylanexedens* YSM1 strain used in this study was isolated and identified according to the method described by Çalık et al. (2017) [37]. Silver nitrate (AgNO₃) and DPPH were purchased from Chem Pure (USA) and Sigma–Aldrich (Germany), respectively. Fetal bovine serum (FBS) and Dulbecco’s Modified Eagle Medium (DMEM) were obtained from Capricorn (Germany).

### Preparation of Postbiotic

The *P. xylanexedens* YSM1 strain was cultivated in Luria-Bertani (LB) broth at 37 °C for 18 h. Following a second activation, the bacterial culture was centrifuged at 6000 rpm for 15 min and washed twice with phosphate-buffered saline (PBS). For standardization and consistency in postbiotic production, the bacterial suspension was adjusted to 0.5 McFarland turbidity (1.5 × 10⁸ cfu/mL). Subsequently, the adjusted culture was inoculated into LB medium at a 2% (v/v) concentration and incubated at 37 °C for 48 h. The resulting postbiotic was produced by centrifuging at 6000 rpm for 30 min, then filtering through a 0.2 μm membrane filter to remove bacterial cells. The clarified postbiotic solution was stored at 4 °C until further use [41, 42].

### Synthesis and Optimization

The synthesis of silver nanoparticles (AgNPs) was optimized using the postbiotic obtained from *P. xylanexedens* YSM1. The optimization process involved varying four parameters: AgNO₃ concentration, AgNO₃ to postbiotic ratio, temperature, and reaction time. First, the effect of silver nitrate (AgNO₃) concentration was tested at concentrations of 1 mM, 3 mM, 5 mM, 7 mM and 10 mM. Next, different AgNO₃ to postbiotic volume ratios (10:1, 5:1, and 1:1) were investigated. The synthesis was then conducted at varying temperatures of 30 °C, 45 °C, and 60 °C to examine the impact of temperature on nanoparticle formation. Finally, the effect of reaction time on synthesis was evaluated by incubating the mixtures for 30–360 min. The formation of nanoparticles was monitored through observation and UV-Vis spectrophotometry, as described in previous studies [11, 43, 44].

### Characterization of AgNPs

The formation of AgNPs was initially confirmed using UV-Vis spectroscopy (Thermo Scientific Genesys 150, USA), which exhibited a characteristic surface plasmon resonance (SPR) peak between 430 and 450 nm. The morphology and size distribution of the synthesized AgNPs were examined using SEM (QUANTA 400 F Field Emission FE-SEM, Philips/FEI, USA). EDX analysis confirmed the presence of silver in the synthesized nanoparticles. The EDX spectrum displayed a prominent peak at 3 keV, which is indicative of silver, thus validating the successful synthesis of AgNPs. FTIR (Vertex 80 FTIR instrument (Bruker, USA)) analysis was conducted to identify the functional groups present in the AgNPs. The crystallinity and phase purity of the synthesized AgNPs were analyzed using XRD (APD 2000 PRO (GNR, Italy) model Cu beam tube XRD Instrument (kV: 40 kV mA: 30 mA), λ=(CoKa) 1.790 Å). The stability of the synthesized AgNPs was evaluated through zeta potential measurements. The hydrodynamic diameter and surface charge of the AgNPs were assessed using Dynamic Light Scattering **(**DLS) (Nano ZS90 (Malvern Instruments, UK)).

### Antimicrobial Activity

The antimicrobial potential of biosynthesized AgNPs was examined against *E. coli* ATCC 25,922, *S. aureus* ATCC 25,923, and *C. albicans* ATCC 10,231. The MIC and MBC were determined for each pathogen. *E. coli* and *S. aureus* were cultured in Mueller-Hinton Broth (MHB), while *C. albicans* was cultured in Sabouraud (2%) Dextrose Broth (SDB) medium for 18 h. The MIC of AgNPs was determined using a broth microdilution assay in 96-well plates, with concentrations ranging from 2000 to 31.25 µg/ml. Pathogens at a McFarland density of 0.5 were inoculated into these media containing AgNPs and incubated at 37 °C for 24 h. After the incubation period, the MIC was established as the concentration that exhibited no visible turbidity. The MBC values were determined by subculturing 10 µl from non-turbid wells onto an agar medium. The resulting growth was recorded after incubation, and the concentration at which 99 − 90% of the initial inoculum was killed was determined. All experiments were performed in triplicate (*n* = 3) [11, 45, 46].

### Antioxidant Activity

The antioxidant activity of bacteria-derived silver nanoparticles was investigated using the DPPH free radical scavenging method. A 0.1 mM DPPH solution was freshly prepared in methanol and mixed with AgNPs samples at concentrations ranging from 1000 to 62.5 to µg/ml. The mixtures were incubated for 30 min in the dark. Following incubation, the absorbance was measured at a wavelength of 517 nm against a blank sample using a UV spectrophotometer. The control sample consisted of the DPPH solution without AgNPs. The percentage removal of DPPH was calculated using the provided formula. Each experiment was independently conducted in triplicate, and the results are presented as mean ± standard deviation (*n* = 3) [11].

### Anti-Biofilm Activity

 The antibacterial activity of biosynthesised AgNPs was evaluated against ***E. coli*** ATCC 25,922, ***S. aureus*** ATCC 25,923, and ***C. albicans*** ATCC 10,231 using a crystal violet-based microtitre plate assay with minor modifications [63, 80, 87]. Briefly, an AgNP stock solution at a concentration of 2000 µg/mL was prepared in Mueller–Hinton Broth (MHB) and homogenised prior to use. A two-fold serial dilution was performed in sterile 96-well flat-bottom microplates to obtain final AgNP concentrations ranging from 1000 to 8.1 µg/mL. Microbial suspensions were prepared from fresh overnight cultures and adjusted to an optical density (OD₆₀₀) of 0.05 at 600 nm. An equal volume of standardised microbial suspension was added to each well containing the diluted AgNPs. The plates were incubated at 37 °C for 24 h under static conditions to allow biofilm formation. After incubation, planktonic cells were carefully removed, and the wells were gently washed three times with sterile PBS solution to remove adherent cells. The remaining biofilms were fixed with 95% methanol for 15 min and air-dried at room temperature. The wells were then stained with a 0.1% (w/v) crystal violet solution for 30 min. Excess dye was removed by rinsing with sterile distilled water, and the bound dye was dissolved using 33% (v/v) acetic acid. The biofilm biomass was quantified by measuring the absorbance at 570 nm using a microplate reader. Wells containing microbial cultures without AgNP were used as controls. The biofilm inhibition percentage was calculated using the following formula. All experiments were conducted in triplicate (*n* = 3).

$$Biofilm\;Inhibition(\%)=((OD_{control}-OD_{treated)/OD\times100}$$ 

### Anticancer Study

The anticancer activity of bacteria-derived silver nanoparticles was evaluated using the MTT assay. This study utilized the human colon adenocarcinoma cell line HT29, with the healthy human lung fibroblast cell line MRC5 serving as a control. Cells were cultured in DMEM medium supplemented with 10% fetal bovine serum (FBS) and 1% penicillin/streptomycin antibiotic in a 5% CO₂ incubator at 37 °C, with medium changes every three days. Cells displaying 80–90% confluence were trypsinized with EDTA and counted using trypan blue staining. The cells were transferred to 96-well plates at a density of 5 × 10³ cells/ml. Various concentrations of AgNPs (500–31.25 µg/ml) were prepared in the cell medium and added to the wells, followed by a 48-hour incubation. At the end of the incubation, the culture medium was removed, and 20 µl of MTT solution was added to each well. After 4 h of incubation at 37 °C, the solutions in the wells were discarded, and 200 µl of DMSO was added to dissolve the stained formazan crystals. Absorbance was measured at 570 nm, with control cells without extract serving as a baseline for calculating percentage viability inhibition. Each experiment was independently conducted in triplicate, and the results are presented as mean ± standard deviation (*n* = 5) [11, 47, 48].

## Results

### Synthesis and Optimization

**Fig. 2:**
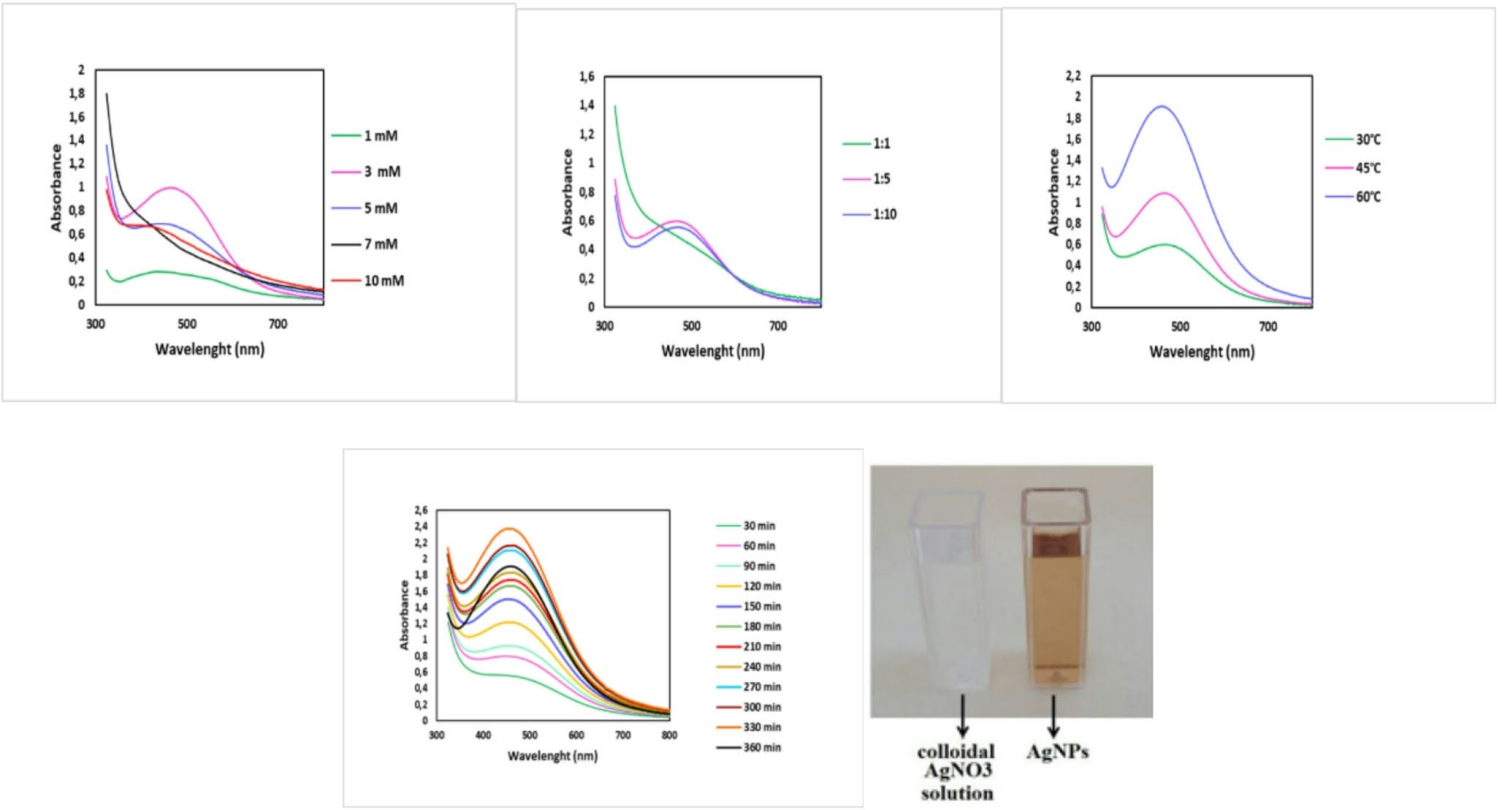
Effect of synthesis parameters on AgNPs formation as monitored by UV–vis spectroscopy: (**a**) AgNO₃ concentration, (**b**) volume ratio of postbiotic to AgNO₃, (**c**) incubation temperature, (**d**) reaction time, and (**e**) the color change from pale yellow to dark brown

The reduction of Ag⁺ ions by the postbiotic derived from *P. xylanexedens* was evidenced by a visible color change in the reaction mixture, transitioning from pale yellow-brown to dark brown (Fig. 2) This shift in color is indicative of silver nanoparticle (AgNP) formation and is attributed to surface plasmon resonance (SPR) phenomena associated with AgNPs, as confirmed by UV–vis spectrophotometric analysis [29 − 23, 43–44]. The color change from pale yellow to dark brown during synthesis indicated the formation of AgNPs. A distinct absorption peak at 435 nm was observed in the UV–Vis spectrum (Fig. 2e).

To optimize the biosynthesis of AgNPs, various parameters were systematically evaluated, including AgNO₃ concentration (1, 3, 5, 7 and 10 mM) (Fig. 2a), the volumetric ratio of AgNO₃ to postbiotic (1:1, 1:5, and 1:10) (Fig. 2b), reaction time (30 to 360 min) (Fig. 2d), and temperature (30, 45, and 60 °C) (Fig. 2c). Among these, 3 mM AgNO₃ yielded the most intense and narrow SPR peak, indicating efficient and stable nanoparticle formation. Varying the AgNO₃:postbiotic ratios revealed that both 1:5 and 1:10 enhanced nanoparticle synthesis compared to 1:1; however, the 1:5 ratio produced slightly sharper peaks and more uniform dispersion and was therefore selected as the optimal condition.

Reaction time had a significant impact on nanoparticle yield. A gradual increase in absorbance intensity was observed with time, and the highest and sharpest SPR peak was recorded at 360 min, suggesting the maximum nanoparticle formation occurred at this point. Temperature optimization studies demonstrated that higher temperatures led to increased absorbance intensity and broader peaks, indicating enhanced synthesis rates but potentially increased polydispersity. The highest synthesis efficiency was achieved at 60 °C, with a well-defined SPR peak and high absorbance, confirming this as the optimal temperature.

Based on these findings, the optimum conditions for AgNPs biosynthesis were determined to be 3 mM AgNO₃ concentration, a 1:5 AgNO₃:postbiotic ratio, 360 min of reaction time, and a reaction temperature of 60 °C. AgNPs synthesized under these optimized parameters were subsequently utilized for detailed characterization and evaluation of their biological activities.

### Characterization

**Fig. 3:**
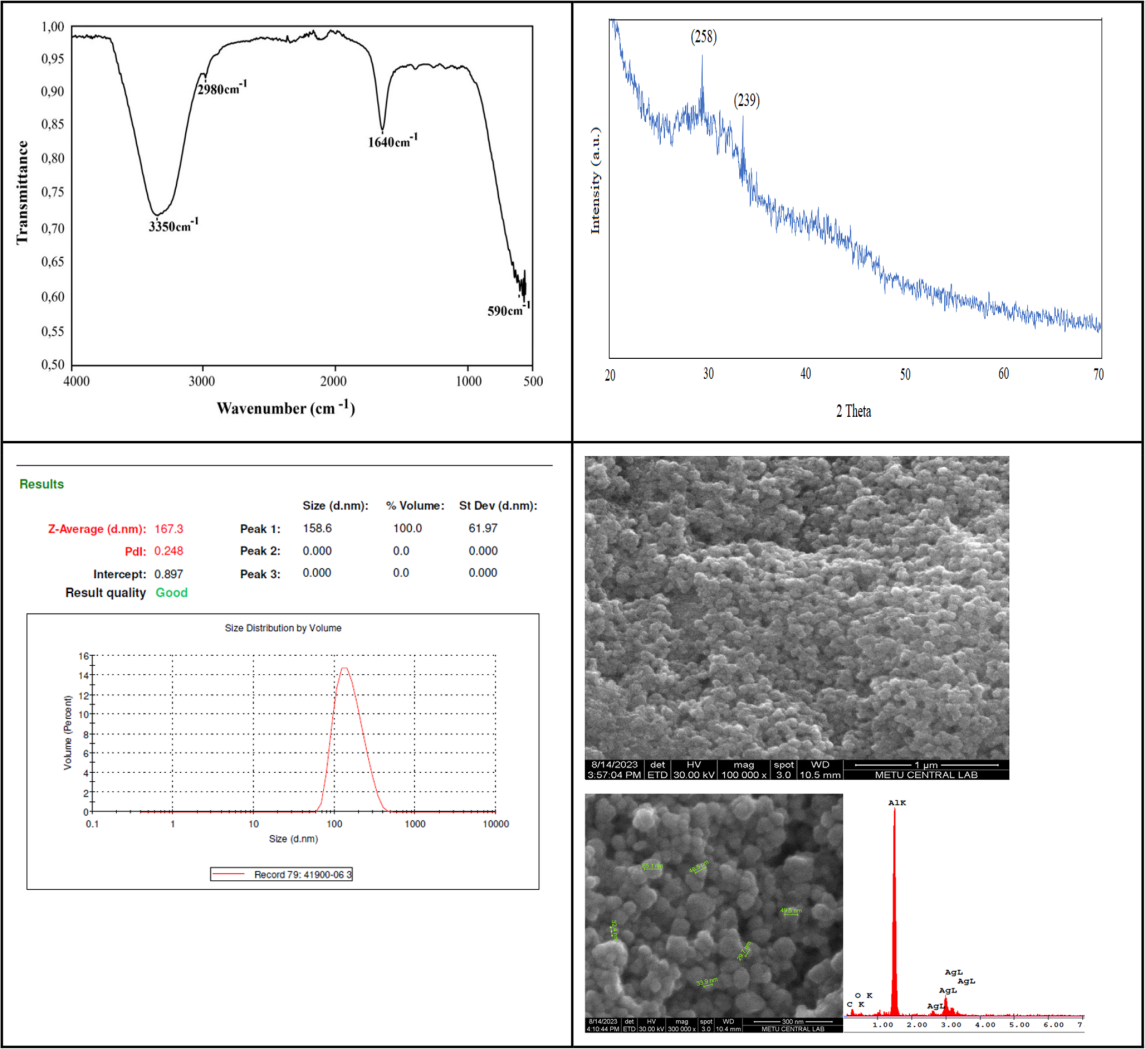
Characterization of silver nanoparticles biosynthesized by *P. xylanexedens.* (**a**) FT-IR spectra indicating functional groups involved in the bioreduction and stabilization of AgNPs (**b**) XRD pattern confirming their crystalline face-centered cubic (FCC) structure, (**c**) Zeta sizer analysis showing hydrodynamic size distribution and colloidal stability, and (**d**) SEM images demonstrating predominantly spherical morphology with The EDX spectrum confirms the presence of silver

FT-IR analysis revealed peaks at 1640 cm⁻¹ (C = O stretching) [12], 2980 cm⁻¹ (C–H stretching), 3350 cm⁻¹ (N–H stretching) [49–51], and 590 cm⁻¹ (C–C deformation), indicating the presence of functional groups associated with amides, aliphatic chains, and proteins (Fig. 3a) [52, 53]. XRD analysis showed characteristic peaks at 2θ values of 29.1° and 31.5°, corresponding to the (259) and (239) planes of the face-centered cubic (FCC) crystal structure of silver (Fig. 3b) [54–56]. SEM images at 50,000× magnification revealed spherical AgNPs with sizes ranging from 29.7 to 65.1 nm. EDX analysis confirmed the presence of elemental silver, with a strong signal observed at 3 keV (Fig. 3d). DLS analysis showed a Z-average hydrodynamic diameter of 167.3 nm and a peak size of 158.6 nm, with a polydispersity index (PDI) of 0.248 (Fig. 3c).

### Antimicrobial Activity of AgNPs against Pathogens

**Fig. 4:**
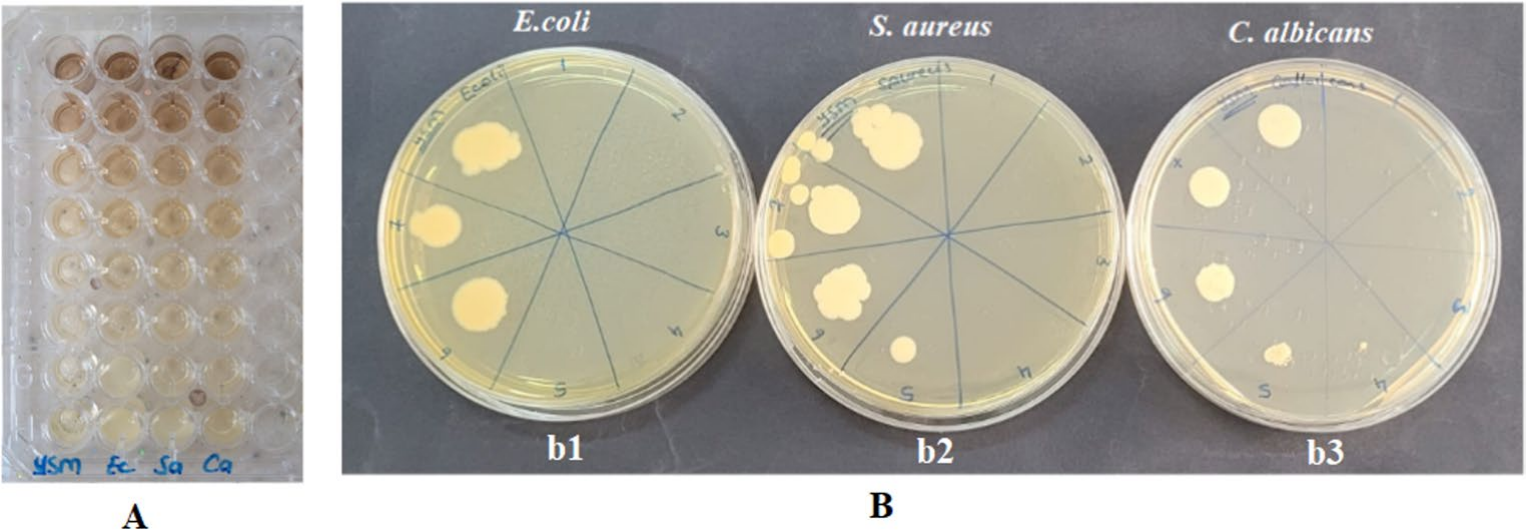
Antimicrobial activity of AgNps (**A**) MIC, (**B**) MBC; b1: *E. coli*, b2: *S. aureus* and b3: *C. albicans*

The biosynthesized AgNPs exhibited notable antimicrobial activity against both Gram-negative (*Escherichia coli*) and Gram-positive (*Staphylococcus aureus*) bacteria, as well as antifungal activity against *Candida albicans*. The antimicrobial efficacy was assessed by determining the minimum inhibitory concentration (MIC) and minimum bactericidal/fungicidal concentration (MBC/MFC) for each pathogen. Among the tested microorganisms, *E. coli* showed the highest sensitivity to AgNPs, with a MIC of 62.5 µg/mL and an MBC of 125 µg/mL. *S. aureus* followed, with a MIC of 125 µg/mL and an MBC of 250 µg/mL. *C. albicans* was the least susceptible, with a MIC of 250 µg/mL and an MFC of 500 µg/mL (Table 2; Fig. 4).

**Table 2:** Antimicrobial activity of AgNPs against pathogens (µg/ml)

	E. coli	S. aureus	C. albicans
MIC	62.5	125	250
MBC	125	250	500

### Anti-Biofilm Activity of AgNPs against Pathogens

**Fig. 5:**
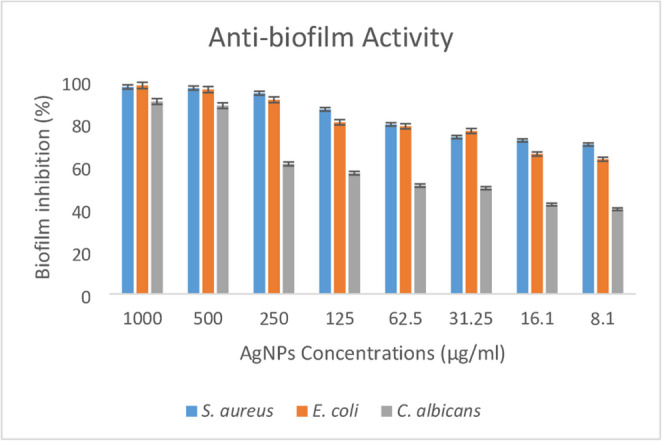
Concentration-dependent inhibition of biofilm formation by postbiotic-mediated AgNPs against *S. aureus*,* E. coli*, and *C. albicans* after 24 h of static incubation, determined using the crystal violet microtiter plate assay. Data are presented as percentage biofilm inhibition relative to untreated controls (mean ± SD, *n* = 3)

The anti-biofilm activity of postbiotic-mediated AgNPs was assessed against ***E. coli***, ***S. aureus***, and ***C. albicans*** using a crystal violet–based microtiter plate assay. The AgNPs exhibited concentration-dependent inhibition of biofilm formation in all tested microorganisms. For ***S. aureus***, strong biofilm inhibition was observed at higher AgNP concentrations, with inhibition values ranging from 94 to 97% at 1000–250 µg/mL. The inhibitory effect decreased to 86.5% at 125 µg/mL and 79.5% at 62.5 µg/mL. Similarly, AgNP treatment effectively suppressed ***E. coli*** biofilm formation. Inhibition rates of 91–98% were recorded at concentrations between 1000 and 250 µg/mL, followed by reductions to 80.5% at 125 µg/mL and 78.6% at 62.5 µg/mL. In contrast, ***C. albicans*** exhibited lower susceptibility to AgNPs. Biofilm inhibition reached 90.2% at 1000 µg/mL and 88.3% at 500 µg/mL, whereas lower inhibition rates were observed at 250 µg/mL (60.9%) and 125 µg/mL (56.7%). Overall, the biosynthesized AgNPs showed concentration-dependent anti-biofilm activity, with bacterial biofilms being more susceptible than the fungal biofilm (Fig. 5).

### Antioxidant Activity of AgNPs

**Fig. 6:**
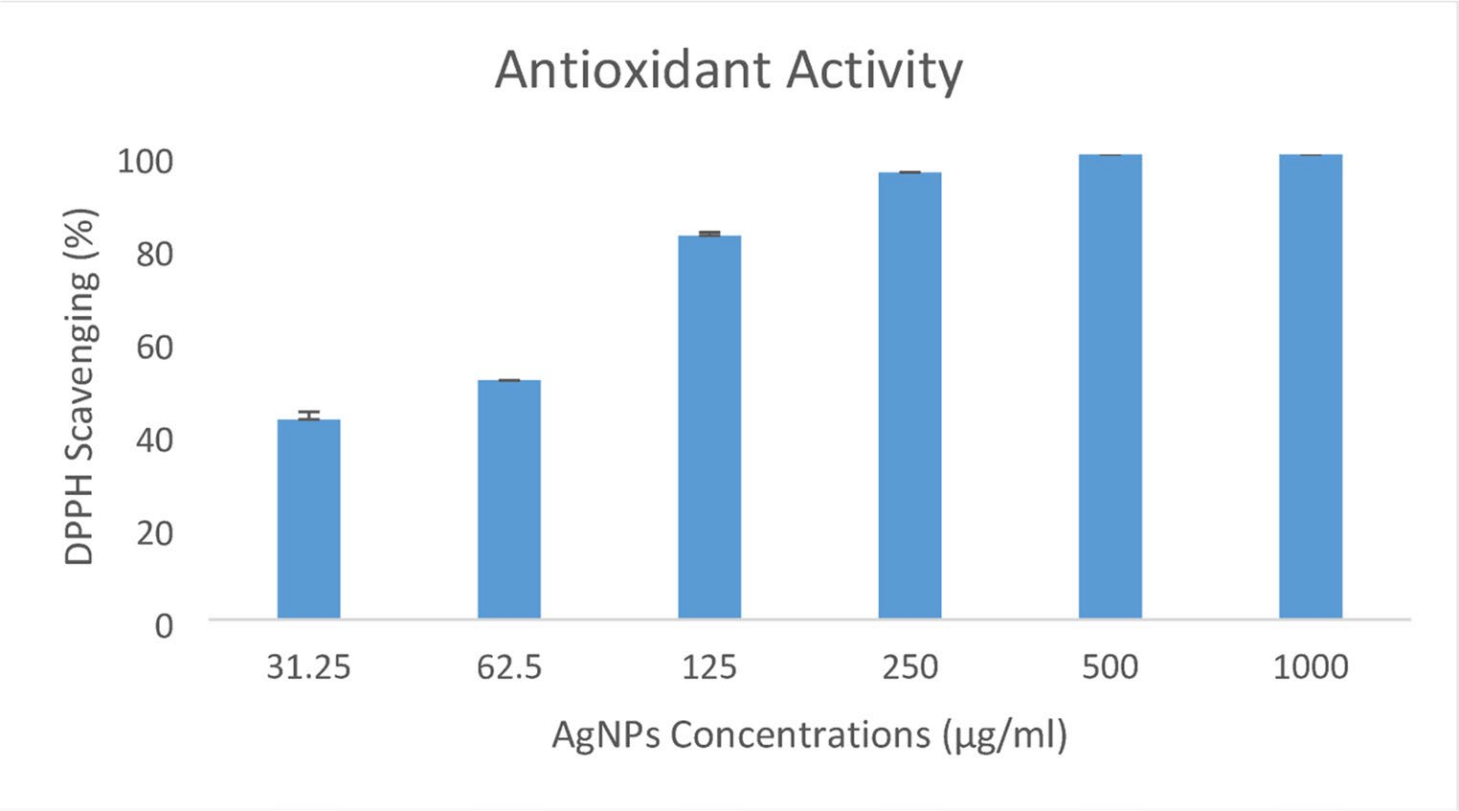
Antioxidant activity assay (DPPH) in AgNPs

The in vitro antioxidant potential of AgNPs synthesized using *P. xylanexedens* YSM1 was evaluated using the DPPH radical scavenging assay. The findings indicated a concentration-dependent enhancement in scavenging efficiency. At a concentration of 62.5 µg/mL, the AgNPs demonstrated an approximate 52% DPPH radical scavenging activity. With increasing concentrations, the radical neutralisation capacity was significantly enhanced, reaching nearly 100% scavenging activity at 500–1000 µg/mL (Fig. 6). The findings suggest that *P. xylanexedens*-mediated AgNPs exhibit notable antioxidant properties, particularly at elevated concentrations.

### Cytotoxic Effects of AgNPs on Cancer Cells

**Fig. 7:**
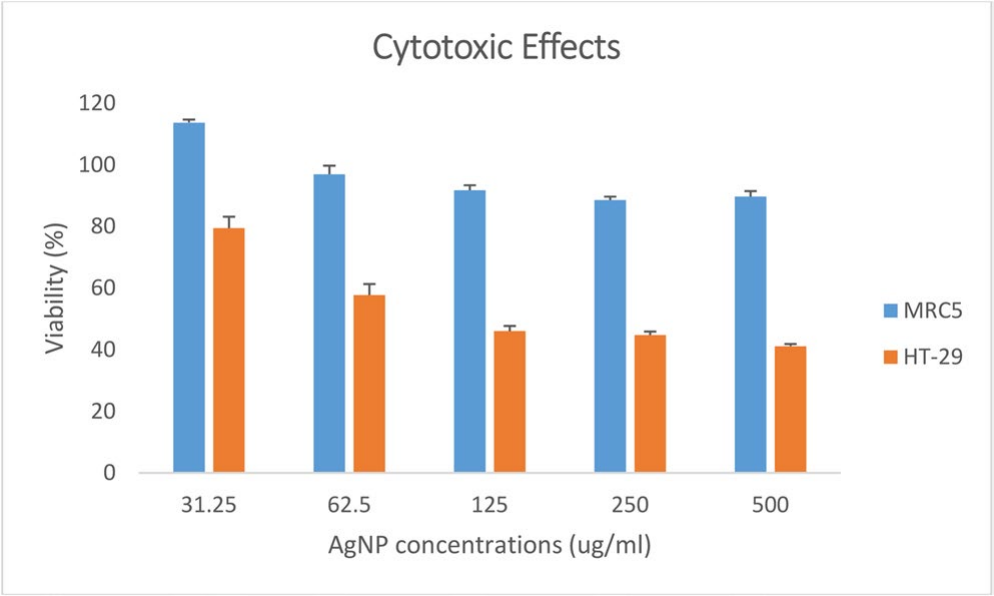
Anticancer activity of AgNPs

The cytotoxic potential of AgNPs synthesized using *P. xylanexedens* YSM1 was evaluated against HT29 human colon adenocarcinoma cells and compared with MRC5 normal lung fibroblast cells using the MTT assay. Cells were treated with AgNPs concentrations ranging from 31,25 to 500 µg/mL (Fig. 7). The results revealed that AgNPs exerted a dose-dependent cytotoxic effect on HT29 cells, with approximately 54% cell death observed at 125 µg/mL, which coincided with the concentration at which complete (100%) DPPH radical scavenging was achieved. In contrast, AgNPs demonstrated limited cytotoxicity toward MRC5 cells (3–10%), indicating a degree of selectivity toward cancer cells.

## Discussion

The global increase in multidrug-resistant (MDR) pathogens and the growing incidence of oxidative stress–related and neoplastic diseases represent major challenges to global health. Conventional antimicrobial and chemotherapeutic strategies are increasingly limited by drug resistance, cytotoxic side effects, and lack of selectivity [7, 23]. Bacteria and cancer cells share similar adaptive mechanisms that promote survival under stress, including oxidative and drug-induced conditions, resulting in parallel resistance pathways. This convergence links microbial dysbiosis, antibiotic resistance, and tumor progression, particularly in colorectal cancer (CRC), where gut microbiota alterations modulate both disease development and treatment response. Moreover, chemotherapy-induced dysbiosis can enhance de novo antimicrobial resistance through SOS-mediated mutations and affect drug efficacy and host toxicity [57, 58]. Therefore, the development of multifunctional agents that can simultaneously combat microbial infections, neutralize oxidative stress, and selectively target cancer cells has become a research priority in nanomedicine [19, 53–55]. In this context, biogenically synthesized AgNPs have emerged as promising candidates due to their broad biological functionality—combining antimicrobial, antioxidant, and anticancer activities in a single nanosystem [9–16].

The microbial synthesis of AgNPs in bacteria generally occurs through two main pathways: intracellular and extracellular biosynthesis [22]– [23]. The intracellular route involves the enzymatic reduction of silver ions within the bacterial cell, primarily mediated by NADPH-dependent nitrate reductase, which converts nitrate to nitrite and transfers electrons to Ag⁺ ions, reducing them to elemental silver (Ag⁰) [24–26]. During this process, nanoparticles can accumulate in the periplasm, cytoplasm, or cell wall due to electrostatic interactions [27, 28]. In contrast, the extracellular mechanism relies on secreted bioactive molecules such as enzymes, proteins, polysaccharides, and secondary metabolites that act as natural reducing and stabilizing agents [12, 29–32, 43]. The bio-reduction process mediated by postbiotic bacterial metabolites offers an additional advantage: it enhances nanoparticle biocompatibility and functionalization while avoiding toxic reagents typically used in chemical synthesis [25, 43, 44, 60]. The present study was designed to explore this multifunctionality by employing the probiotic *P. xylanexedens* YSM1 as a biological platform for an extracellular, postbiotic-mediated AgNPs synthesis. This approach aimed to address two critical biomedical problems—(i) the urgent need for effective antimicrobial agents against pathogenic microorganisms and (ii) the search for selective anticancer strategies with minimal toxicity toward normal cells.

In the present study, an extracellular, postbiotic-mediated biosynthesis approach was adopted for the first time using *P. xylanexedens* YSM1. This method offers several advantages over intracellular synthesis, including ease of nanoparticle recovery, scalability, and reduced contamination risk. Moreover, postbiotics—comprising metabolites and bioactive molecules derived from probiotic cultures—serve as efficient reducing and capping agents, enhancing nanoparticle stability and biocompatibility while eliminating the need for toxic chemical reagents [12, 25, 43, 60]. These attributes make postbiotic-mediated extracellular synthesis a sustainable and safe alternative for producing multifunctional AgNPs suitable for biomedical applications. A distinct color transition from pale yellow to dark brown confirmed nanoparticle formation, corresponding to a surface plasmon resonance (SPR) peak at 435 nm in the UV–Vis spectrum (Fig. 2). Similar studies using *Paenibacillus* species have reported characteristic SPR peaks between 410 and 450 nm [61–65], indicating strong agreement with our findings. Optimization experiments were performed based on UV–Vis spectral analyses to identify the most effective synthesis conditions. Reaction parameters such as temperature, AgNO₃ concentration, and incubation time are known to influence the position and intensity of SPR peaks, which reflect nanoparticle formation efficiency [66–70]. The temperature can significantly affect the formation and growth, the shape, size, and size distribution of particles [7, 65]. Thermal acceleration of nucleation and growth with increasing temperature has been widely documented in green and biological syntheses of AgNPs. However, extreme temperatures (e.g., > 80 °C) can reportedly denature proteins and alter nucleation pathways, resulting in larger, aggregated particles and reduced colloidal stability [66, [67]. Similarly, related studies report a clear positive correlation between AgNO₃ concentration and average particle/crystallite size [60, 70]. The time-dependent increase in nanoparticle synthesis is known. Time-dependent redshifts in the SPR are a widely used indicator of particle growth and aggregation in green synthesis reports [11, 71]. Accordingly, the optimized parameters were determined as 3 mM AgNO₃ concentration, 1:5 AgNO₃-to-postbiotic ratio, 360 min reaction time, and 60 °C incubation temperature. These conditions provided a strong and stable SPR signal, indicating efficient nanoparticle formation under biologically favorable conditions.

The biosynthesized AgNPs were characterized using multiple analytical techniques, including UV–Vis spectroscopy, FT-IR, XRD, DLS, and SEM–EDX, to elucidate their optical, structural, and morphological properties. In the FT-IR spectra (Fig. 3a), several significant absorbance peaks were observed, which correspond to various functional groups involved in the synthesis and stabilization of AgNPs. The absorbance at 1640 cm⁻¹ is attributed to the stretching vibration of the carbonyl (C = O) group, indicating the presence of amides or carboxylates [16]. C–H stretching vibration was observed at 2980 cm⁻¹, which is typically associated with aliphatic hydrocarbons [49]– [50]. A narrow peak at 3350 cm⁻¹ corresponds to the presence of an –NH stretching vibration, indicative of amide groups, suggesting that amine groups may be involved in the stabilization of AgNPs [51]. Additionally, a peak at 590 cm⁻¹ can be attributed to C–C deformation vibrations, which are commonly associated with the structural components of proteins [52, 53]. These results suggest that the carboxyl (C = O) and amine (–NH) groups from the bacterial proteins may play a crucial role in the reduction of silver ions and the stabilization of the synthesized silver nanoparticles [72–74]. Similar FT-IR spectral features have been reported in *Paenibacillus*-mediated AgNPs, confirming that proteins, carboxylic acids, and polysaccharides secreted extracellularly act as both reducing and capping agents [62–64]. The presence of strong bands at 3350–3450 cm⁻¹ (O–H/N–H), 1640–1650 cm⁻¹ (amide I), and 1400–1380 cm⁻¹ (COO⁻) is consistent with previously characterized Paenibacillus-based AgNPs, suggesting the involvement of proteins and phenolic compounds in nanoparticle stabilization [60, 65]. These findings indicate that the postbiotic metabolites of *P. xylanexedens* not only mediate the reduction of Ag⁺ ions but also enhance nanoparticle stability by acting as natural biocapping agents, consistent with prior reports on biogenic AgNPs synthesized by *Paenibacillus* species. These functional groups likely serve as capping agents, ensuring the colloidal stability of the AgNPs in solution and preventing aggregation. Moreover, these biomolecular coatings may enhance the biological performance of AgNPs by improving their surface functionality and facilitating interactions with microbial membranes or reactive oxygen species, thus linking the physicochemical characteristics of the nanoparticles with their antimicrobial and antioxidant activities [62–65].

The X-ray diffraction (XRD) pattern (Fig. 3b) of the biosynthesized AgNPs exhibited distinct diffraction peaks at 2θ = 29.1° and 31.5°, corresponding to the (259) and (239) planes of the face-centered cubic (FCC) crystal structure of metallic silver. The presence of these peaks confirms that the AgNPs synthesized using *P. xylanexedens* YSM1 possess a highly crystalline nature and well-defined lattice arrangement [54–56]. Similar XRD diffraction patterns have been reported for AgNPs synthesized using *Paenibacillus* strains, with characteristic reflections typically observed around 38°, 44°, 64°, and 77°, indexed to the (111), (200), (220), and (311) planes of FCC silver [62–64]. The slight variation in peak positions observed in the present study may be attributed to the interaction of bioactive compounds from the *P. xylanexedens* postbiotic matrix with the nanoparticle surface, which can influence lattice strain and particle size. The well-resolved and intense diffraction peaks further indicate the high purity and crystalline quality of the AgNPs, suggesting that the extracellular postbiotic-mediated synthesis pathway successfully facilitated the reduction of Ag⁺ ions and stabilization of uniform nanoparticles without significant amorphous background. This crystalline order is consistent with previous findings that biologically synthesized AgNPs generally exhibit FCC geometry and nanoscale crystallite dimensions (typically 20–60 nm), in agreement with the TEM results [61–63].

The morphology of the biosynthesized AgNPs was examined using scanning electron microscopy (SEM), which revealed that the nanoparticles were uniformly dispersed over a wide surface area without apparent aggregation (Fig. 3d). SEM micrographs at 300,000× magnification showed that the AgNPs were predominantly spherical in shape, with sizes ranging from 29 to 65 nm, consistent with the nanoscale features observed in other *Paenibacillus*-mediated AgNP syntheses [62, 63]. The relatively uniform morphology suggests effective capping and stabilization by biomolecules secreted in the *P. xylanexedens* postbiotic filtrate, which prevented particle coalescence during nucleation and growth. Energy-dispersive X-ray (EDX) spectroscopy confirmed the elemental composition of the nanoparticles. A strong and sharp characteristic signal at approximately 3 keV corresponded to the silver (Ag) Lα emission, validating the formation of metallic silver nanoparticles (Fig. 3d). In addition to silver, minor signals for carbon and oxygen were also observed, which can be attributed to the presence of organic residues from the bacterial metabolites acting as capping agents. Similar EDX profiles have been reported for biosynthesized AgNPs, supporting the role of bacterial biomolecules in nanoparticle stabilization and surface modification [63, 64]. Overall, the SEM and EDX analyses corroborate the successful extracellular biosynthesis of well-dispersed, crystalline, and biocapped AgNPs. These morphological and elemental characteristics are consistent with the results of the UV–Vis, FT-IR, and XRD analyses, collectively confirming the formation of stable and biocompatible AgNPs through the postbiotic-mediated reduction process.

Dynamic Light Scattering (DLS) analysis was conducted to evaluate the hydrodynamic diameter, size distribution, and colloidal stability of the biosynthesised AgNPs. The Z-average diameter was determined to be 167.3 nm, while the peak particle size distribution was centred at 158.6 nm (Fig. 3c). The polydispersity index (PDI) was calculated as 0.248, indicating a moderately narrow size distribution and acceptable homogeneity within the nanoparticle population. Notably, the particle size obtained from DLS analysis was higher than that measured by SEM. This discrepancy is expected, as DLS measures the hydrodynamic diameter of nanoparticles in suspension, which includes not only the metallic core but also the surrounding capping biomolecules and solvation layer [71]. In contrast, SEM provides the dry-state physical size of the metallic core, often resulting in smaller measured diameters. Similar findings have been reported in studies of *Paenibacillus*-mediated AgNPs, where DLS-measured sizes were significantly larger than TEM/SEM data due to the presence of organic coatings derived from proteins and polysaccharides acting as stabilising agents [62–64]. Overall, the relatively low PDI value suggests a stable and well-dispersed colloidal suspension, confirming that the biomolecules secreted by *P. xylanexedens* postbiotics effectively prevented nanoparticle aggregation and ensured long-term dispersion stability in aqueous media.

AgNPs act through multiple, often convergent antimicrobial mechanisms that collectively reduce the likelihood of resistance development. Mechanistically, AgNPs (i) physically interact with and disrupt microbial membranes, causing increased permeability and leakage of intracellular contents; (ii) release Ag⁺ ions that bind to thiol groups in enzymes and structural proteins, impairing respiration and metabolic processes; (iii) interact with nucleic acids to hinder replication and transcription; and (iv) catalyze intracellular reactive oxygen species (ROS) formation, leading to oxidative damage of lipids, proteins, and DNA [57, 66, 75, 76]. In the present study, silver nanoparticles synthesized using the postbiotic of *P. xylanexedens* YSM1 exhibited potent antimicrobial activity against both bacterial and fungal pathogens (Fig. 4). Among the tested organisms, *E. coli* showed the highest susceptibility (MIC: 62.5 µg/mL; MBC: 125 µg/mL), followed by *S. aureus* (MIC: 125 µg/mL; MBC: 250 µg/mL), while *C. albicans* displayed the lowest sensitivity (MIC: 250 µg/mL; MFC: 500 µg/mL). This gradient in susceptibility reflects intrinsic structural differences among microbial cell walls—Gram-negative bacteria, with thinner peptidoglycan layers and LPS-rich outer membranes, are typically more permeable to AgNP penetration than Gram-positive or fungal cells [24, 25]. Comparable antimicrobial profiles have been reported for other *Paenibacillus*-mediated AgNPs. For instance, *Paenibacillus sp.*–derived AgNPs demonstrated strong antibacterial activitie against *E. coli* [62]; *P. polymyxa* exopolysaccharide–capped AgNPs exhibited significant inhibition of *E. coli* and *S. aureus* at 75–150 µg/mL [65]; *Paenibacillus* isolates from aquatic environments yielded AgNPs with potent antibiofilm effects against *E. coli* [63]; and diverse *Paenibacillus* strains contributed to AgNPs showing broad antibacterial and antifungal efficacy against drug-resistant pathogens [64]. Collectively, these findings indicate that *Paenibacillus* species—owing to their rich secretome of extracellular enzymes, redox-active peptides, and polysaccharides—provide a favorable biochemical milieu for nanoparticle bioreduction and stabilization. The antimicrobial activity of the biosynthesized AgNPs can be attributed not only to well-established mechanisms such as Ag⁺ ion release, ROS generation, and membrane disruption but also to the physicochemical characteristics developed during green synthesis. FT-IR analysis revealed the presence of hydroxyl, carbonyl, and amide groups originating from postbiotic metabolites, which likely participated in both the reduction of Ag⁺ ions and the stabilization of nanoparticle surfaces. These biomolecules may contribute to colloidal stability and influence Ag⁺ ion interaction with microbial cells, thereby supporting the observed antimicrobial effects [81–83].

Biofilm-associated infections constitute one of the most critical drivers of antimicrobial resistance, as microorganisms embedded within biofilms can tolerate antimicrobial concentrations far exceeding those effective against their planktonic counterparts. This heightened tolerance arises from multiple factors, including limited antimicrobial penetration, altered metabolic states, activation of stress-response pathways, and enhanced horizontal gene transfer within the biofilm matrix [80–83]. Consequently, biofilms formed by clinically relevant pathogens such as ***Staphylococcus aureus***, ***Staphylococcus epidermidis***, ***Escherichia coli***, ***Pseudomonas aeruginosa***, ***Salmonella typhimurium***, ***Streptococcus mutans***, ***Klebsiella pneumoniae***, and ***Candida albicans*** are strongly associated with persistent infections, medical device contamination, and therapeutic failure. These challenges underscore the urgent need for alternative strategies capable of disrupting both biofilm architecture and resistance mechanisms [80–85]. The concordance between planktonic antimicrobial activity and biofilm inhibition has been widely regarded as a desirable feature for nanomaterials intended to mitigate persistent and device-associated infections. Recent studies have demonstrated that nanostructured systems can effectively disrupt biofilm architecture, increase membrane permeability, and interfere with quorum sensing pathways in drug-resistant pathogens, including ESKAPE-associated bacteria [84, 87–92]. Moreover, accumulating evidence indicates that green-synthesized metallic nanoparticles—such as silver, iron, titanium, selenium, and magnesium nanoparticles—exhibit enhanced antibiofilm performance by combining high surface reactivity with sustained antimicrobial action [82–88]. In this context, El Shanshoury et al. (2023) reported that AgNPs synthesized using a ***Paenibacillus*** HSHPH isolateexhibited high antibiofilm efficacy against ***E. coli*** ATCC 11,922 at a concentration of 32 µg/mL [63]. Similarly, Abishad et al. (2022) demonstrated that ***Lactobacillus acidophilus***–derived AgNPs effectively inhibited biofilm formation in multidrug-resistant enteroaggregative ***E. coli*** (MDR-EAEC) strains [80]. More recently, Jeyachandran et al. (2025) showed that ***Bacillus lentus***–mediated AgNPs (50 µg/mL) reduced extracellular polymeric substance (EPS) production by approximately 50% in ***Bacillus licheniformis*** and ***P. aeruginosa***, further supporting the antibiofilm potential of biologically synthesized AgNPs [83]. In the present study, the antibiofilm activity of postbiotic-mediated AgNPs synthesized using ***Paenibacillus xylanexedens*** YSM1 was evaluated against ***S. aureus***, ***E. coli***, and ***C. albicans*** using a crystal violet–based microtiter plate assay. Although no internationally standardized guideline exists for the quantitative interpretation of antibiofilm assays, many studies classify inhibition efficiency based on percentage reduction relative to untreated controls, commonly defining ≥ 75% inhibition as strong antibiofilm activity, 50–75% as high, 25–50% as moderate, and < 25% as low inhibition. According to this widely adopted framework, the biosynthesized AgNPs exhibited strong antibiofilm activity against both ***S. aureus*** and ***E. coli*** at concentrations ≥ 62.5 µg/mL, with inhibition rates exceeding 90% at higher concentrations. In contrast, ***C. albicans*** biofilms required higher AgNP concentrations (≥ 500 µg/mL) to achieve comparable inhibition, indicating a relatively reduced susceptibility. The observed concentration-dependent antibiofilm profile is consistent with the known structural and compositional differences between bacterial and fungal biofilms. Bacterial biofilms are primarily composed of extracellular polysaccharides, proteins, and extracellular DNA, whereas ***C. albicans*** biofilms possess a more complex extracellular matrix enriched with β-glucans, mannans, and chitin, which confers enhanced tolerance to antimicrobial agents [1, 80, 83, 84]. Notably, the antibiofilm-effective concentrations largely overlapped with the MIC values obtained in planktonic antimicrobial assays, particularly for ***E. coli*** and This overlap indicates a coherent antimicrobial–antibiofilm relationship**,** suggesting that postbiotic-mediated AgNPs retain biological activity against both free-living and surface-associated microbial states. Within this framework, the strong antibiofilm activity observed for ***P. xylanexedens***–derived AgNPs highlights the relevance of postbiotic-mediated synthesis strategies. The presence of bioactive metabolites on the nanoparticle surface, as confirmed by FT-IR analysis, may facilitate interactions with extracellular polymeric substances and microbial membranes, thereby contributing to effective biofilm inhibition. Collectively, these findings support the potential of postbiotic-derived AgNPs as multifunctional nanomaterials capable of targeting both planktonic and biofilm-associated microbial populations—a feature of particular importance in addressing antimicrobial resistance and biofilm-related infections.

In addition to their antimicrobial efficacy, AgNPs also exhibit notable antioxidant potential, which plays a vital role in mitigating oxidative stress–related cellular damage. The DPPH assay assesses the ability of a compound to donate electrons or hydrogen atoms to neutralize stable DPPH radicals, leading to a measurable decline in absorbance. Two major mechanisms are involved in this process—single electron transfer (SET) and hydrogen atom transfer (HAT)—with their relative contribution depending on the redox chemistry of the reducing agents. In this study, the biosynthesized AgNPs demonstrated concentration-dependent DPPH radical scavenging activity, increasing from approximately 52% at 62.5 µg/mL to nearly 100% at 500–1000 µg/mL (Fig. 6). This strong antioxidant response can be attributed to bioactive postbiotic molecules (proteins, phenolics, and polysaccharides) acting as capping and stabilizing agents, which contribute additional electron-donating functionality and enhance redox reactivity [16, 24]. The FT-IR spectra support this interpretation by confirming the presence of hydroxyl, carbonyl, and amide functional groups associated with these postbiotic metabolites. Comparable dose-dependent scavenging trends have been widely reported for green- and probiotic-mediated AgNPs, particularly those synthesized using *Lactobacillus* and *Bacillus* species [63, 76–79]. However, to the best of our knowledge, this is among the first reports demonstrating DPPH radical scavenging activity for *Paenibacillus*-derived AgNPs. This observation expands the known functional repertoire of *Paenibacillus*-based biogenic nanoparticles, suggesting that postbiotic components from this genus confer not only antimicrobial but also significant redox-regulating potential. Given the established link between oxidative stress, inflammation, and tumor progression, such dual antimicrobial–antioxidant functionality positions these AgNPs as promising candidates for therapeutic and biomedical applications.

Cancer remains one of the leading causes of mortality worldwide, accounting for nearly 10 million deaths each year, with CRC ranking second in cancer-related deaths and third in incidence [58 , 59, 93]. Recent metatranscriptomic studies have shown that the gut microbiota in CRC patients undergoes not only compositional but also functional alterations under oxidative and inflammatory stress, characterized by the increased activity of ESCAPE/ESKAPE pathogens (*Enterococcus faecium*,* Staphylococcus aureus*,* Clostridium difficile/Klebsiella pneumoniae*,* Acinetobacter baumannii*,* Pseudomonas aeruginosa*, and *Enterobacteriaceae*) and the upregulation of antibiotic resistance genes [57, 58]. These dysbiotic and inflammatory conditions, particularly in immunocompromised patients undergoing chemotherapy or radiotherapy, are known to exacerbate multidrug resistance, increase infection susceptibility, and impair treatment efficacy. Indeed, chemotherapy- or radiotherapy-induced immunosuppression can elevate the risk of opportunistic infections caused by MDR Gram-negative bacteria, with mortality rates reported between 60% and 84% [57]. In this context, nanomaterials exhibiting both antimicrobial and anticancer activities—such as the green-synthesized AgNPs developed in this study—represent promising multifunctional agents. Their combined effects may help mitigate both tumor progression and infection risk within the oxidative and dysbiotic microenvironments characteristic of CRC and its treatment. Previous reports have demonstrated the anticancer potential of green-synthesized AgNPs against colon cancer cell lines such as SW480 [94], HCT116 [94–98], HT-29 [22, 98] and Caco-2 [99], showing concentration-dependent cytotoxicity with varying IC₅₀ values depending on the biological source and synthesis route. For instance, AgNPs synthesized using *Paenibacillus sp.* exhibited an IC₅₀ value of 81.54 µg/mL against HCT116 cells [58], while *P. polymyxa*–derived AgNPs showed cytotoxicity toward SK-MEL melanoma cells, with some degree of toxicity to normal epithelial cells [61]. In the present study, postbiotic-derived AgNPs were evaluated for their cytotoxic activity against human colorectal adenocarcinoma (HT-29) and normal lung fibroblast (MRC-5) cell lines using the MTT assay in accordance with ISO 10993-5 guidelines. According to these criteria, reductions in cell viability of ≥ 50%, 21–50%, 11–20%, and ≤ 10% are interpreted as highly cytotoxic, moderately cytotoxic, slightly cytotoxic, and non-cytotoxic effects, respectively [100]. Treatment with 125 µg/mL AgNPs led to a greater than 54% reduction in HT-29 cell viability, indicating a strong cytotoxic effect, whereas MRC-5 cells maintained approximately 92% viability, suggesting non-cytotoxic behavior toward normal fibroblasts (Fig. 7). These findings indicate a degree of selectivity, with malignant cells being more susceptible to postbiotic-mediated AgNPs than healthy cells. Considering the established interplay between microbial dysbiosis, oxidative stress, and tumor progression, it is plausible that *P. xylanexedens*–derived AgNPs exert their effects through redox-related pathways that impact both microbial and cancer cell metabolism. By combining antimicrobial, antioxidant, and selective anticancer activities within a single nanosystem, these biologically synthesized AgNPs provide a potential platform for integrative therapeutic applications aimed at restoring microbial balance and improving clinical outcomes in CRC management.

Taken together, these findings demonstrate that postbiotic-mediated AgNPs derived from probiotic *P. xylanexedens* YSM1 exhibit synergistic biological activities, encompassing broad-spectrum antimicrobial effects, strong antioxidant potential, and selective anticancer properties. The results emphasize the potential of probiotic-based nanotechnology as a multifunctional therapeutic strategy that bridges infection control, oxidative stress management, and cancer therapy within a single biocompatible nanoplatform.

### Performance Evaluation of the Study

This study presents a significant contribution to the field of sustainable nanotechnology, highlighting the use of a novel bacterial strain, the multifaceted investigation of biological activities, and the potential of (AgNPs) synthesized through bacterial processes in biomedical applications. The performance evaluation of the study has been combined with the findings of the SWOT analysis to provide a comprehensive view of the research’s impact.


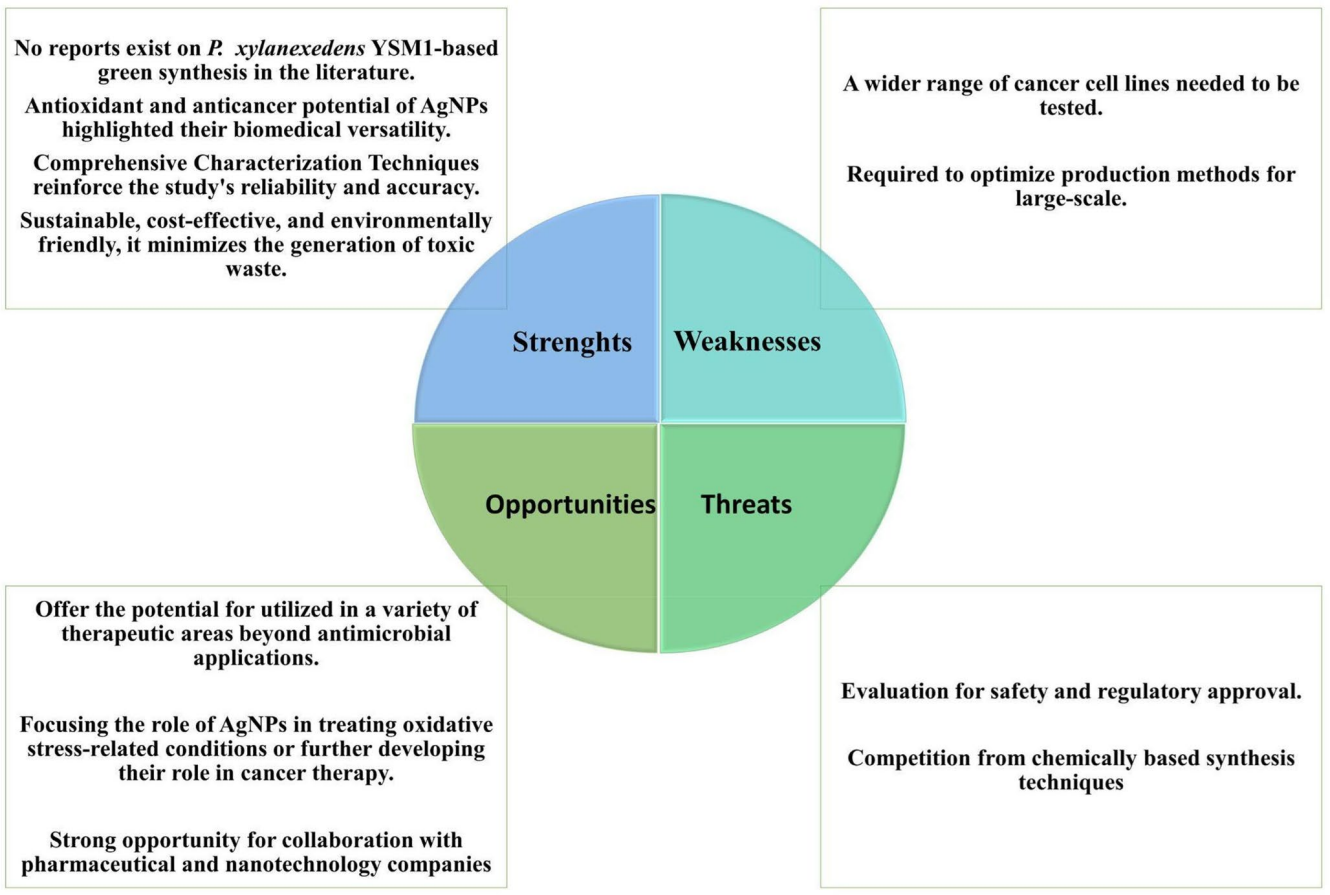


## Conclusions

This study demonstrated that postbiotic-mediated synthesis using *Paenibacillus xylanexedens* YSM1 provides an efficient, environmentally benign approach for the production of stable, crystalline, and biocompatible AgNPs. Comprehensive characterization confirmed that functional biomolecules such as hydroxyl, carbonyl, and amide groups from postbiotic metabolites played a key role in the bioreduction and stabilization of AgNPs. The synthesized nanoparticles showed concentration-dependent antioxidant activity and broad-spectrum antimicrobial, and anti-biofilm effects, particularly against *E. coli*, *S. aureus*, and *C. albicans* comparable to previously reported *Paenibacillus*-derived AgNPs. Moreover, the AgNPs exhibited selective cytotoxicity toward colorectal adenocarcinoma (HT-29) cells, while maintaining high viability in normal fibroblasts, indicating a degree of biological selectivity. These combined antimicrobial, antioxidant, and selective anticancer properties suggest that *P. xylanexedens*–mediated AgNPs may serve as a multifunctional nanomaterial with potential applications in infection control and oxidative stress–associated conditions, including cancer-related microbial dysbiosis. Further mechanistic studies and in vivo validation are warranted to better elucidate their safety profile, therapeutic range, and interaction with host–microbiome systems.

### Challenges and Future Perspective

Despite these promising findings, several challenges remain to be addressed before clinical or industrial translation can be considered. First, although in vitro assays demonstrated selective cytotoxicity toward cancer cells with minimal effects on normal fibroblasts, comprehensive in vivo toxicity, biodistribution, and long-term safety studies are required to fully evaluate biocompatibility. Second, while the antibiofilm activity was robust against ***S. aureus*** and ***E. coli***, broader evaluation against multispecies biofilms and clinically isolated multidrug-resistant strains would strengthen the translational relevance of these findings. In addition, the molecular interactions between postbiotic-derived surface functional groups and microbial or cancer cell targets remain to be elucidated in greater mechanistic detail.

Future research should focus on elucidating the precise role of postbiotic components in nanoparticle functionalization, stability, and biological targeting. Investigations into synergistic combinations of postbiotic-mediated AgNPs with conventional antimicrobials or chemotherapeutics may further enhance efficacy while reducing required dosages. Moreover, expanding applications toward biofilm-resistant medical coatings, wound dressings, and microbiota-modulating nanotherapeutics represents a promising direction. Overall, postbiotic-mediated AgNPs derived from ***P. xylanexedens*** offer a sustainable and multifunctional nanoplatform with significant potential to address antimicrobial resistance, biofilm-associated infections, oxidative stress, and cancer-related challenges, warranting further translational and mechanistic investigations.

## Data Availability

Not applicable.

## References

[1] Varshan GA, Namasivayam SKR (2025) A critical review on sustainable formulation of anti-quorum sensing compounds using nanotechnology principles against Candida albicans. BioNanoScience 15(1):161. 10.1007/s12668-024-01685-6

[2] Lavanya M, Namasivayam SKR, John A (2024) Developmental formulation principles of food preservatives by nanoencapsulation—fundamentals, application, and challenges. Appl Biochem Biotechnol 196(10):7503–7533. 10.1007/s12010-024-04943-138713338 10.1007/s12010-024-04943-1

[3] Lavanya M, Namasivayam SKR (2025) Eco-friendly fabrication of magnesium oxide nanoparticles from *Clitoria ternatea* and their influence on plant growth parameters of *Vigna mungo*, soil nutrient dynamics and computational analysis. Plant Nano Biology 100200. 10.1016/j.plana.2025.100200

[4] Raj LA, Annushrie A, Namasivayam SKR (2025) Anti bacterial efficacy of photo catalytic active titanium di oxide (TiO2) nanoparticles synthesized via green science principles against food spoilage pathogenic bacteria. The Microbe 7:100331

[5] Reddy AS, Chen CY, Chen CC, Jean JS, Chen HR, Tseng MJ, Fan CW, Wang JC (2010) Biological synthesis of gold and silver nanoparticles mediated by the bacteria *Bacillus subtilis*. J Nanosci Nanotechnol 10:6567–6574. 10.1166/jnn.2010.251921137763 10.1166/jnn.2010.2519

[6] Ahamad I, Nadeem M, Rizvi MMA, Fatma T (2025) Bio-fabricated silver nanoparticles: therapeutic evaluation as a potential nanodrug against cervical and liver cancer cells. Discover Nano 20(1):47. 10.1186/s11671-025-04212-y40000514 10.1186/s11671-025-04212-yPMC11861485

[7] Hawsawi NM, Hamad AM, Rashid SN, Alshehri F, Sharaf M, Zakai SA, Yousef SAA, Ali AM, Abou-Elnour A, Alkhudhayri A, Elrefaei NG, Elkelish A (2023) Biogenic silver nanoparticles eradicate of *Pseudomonas aeruginosa* and Methicillin-resistant *Staphylococcus aureus* (MRSA) isolated from the sputum of COVID-19 patients. Front Microbiol 14:1142646. 10.3389/fmicb.2023.114264637143540 10.3389/fmicb.2023.1142646PMC10153441

[8] El Dougdoug NK, Attia MS, Malash MN, Abdel-Maksoud MA, Malik A, Kiani BH, Fesal AA, Rizk SH, El-Sayyad GS, Harb N (2025) *Aspergillus fumigatus*-induced biogenic silver nanoparticles’ efficacy as antimicrobial and antibiofilm agents with potential anticancer activity: an in vitro investigation. Microb Pathog 199:106950. 10.1016/j.micpath.2024.10695039303958 10.1016/j.micpath.2024.106950

[9] Alex AM, Subburaman S, Chauhan S, Ahuja V, Abdi G, Tarighat MA (2024) Green synthesis of silver nanoparticle prepared with *Ocimum species* and assessment of anticancer potential. Sci Rep 14(1):11707. 10.1038/s41598-024-61946-y38777818 10.1038/s41598-024-61946-yPMC11111742

[10] Helmlinger J, Sengstock C, Groß-Heitfeld C, Mayer C, Schildhauer TA, Köller M, Epple M (2016) Silver nanoparticles with different size and shape: equal cytotoxicity, but different antibacterial effects. RSC Adv 6(22):18490–18501. 10.1039/C5RA27836H

[11] Doğan SY, Kaya S, Solak EK (2024) Green synthesis of silver nanoparticles using *Origanum onites* extract: effect of temperature and time on antioxidant and antimicrobial activity. J Biomed Res Environ Sci 5(9):1214–1228. 10.37871/jbres2009

[12] Thanuja KJ, Vaishnavi R, Ragunathan R, Johney J (2025) Biogenic synthesis of silver nanoparticles using secondary metabolites of Lactiplantibacillus plantarum and its potential applications. J Drug Delivery Ther, *15*(9)

[13] Bodur OC, Keskin M, Keskin Ş, Arslan F (2025) Green synthesis of *Ananas comosus* (L.) Merr. based-silver nanoparticles and determination of their potential in electrochemical detection of H2O2. Biomass Conver Biorefin 1–11. 10.1007/s13399-025-06823-y

[14] Albao MJF, Calsis JRF, Dancel JO, De Juan-Corpuz LM, Corpuz RD (2025) Silver nanoparticle‐infused hydrogels for biomedical applications: a comprehensive review. J Chin Chem Soc 72(2):124–162. 10.1002/jccs.202400303

[15] Pathan R, Vidyavathi M, Kumar RS, Narasimha G (2025) Design, development, and evaluation of silver nanoparticles synthesized via green technology for antioxidant, antimicrobial, and anticancer activities. BioNanoSci 15:255. 10.1007/s12668-025-01852-3

[16] Al-asbahi MGSS, Al-Ofiry BA, Saad FAA, Alnehia A, Al-Gunaid MQA (2024) Silver nanoparticles biosynthesis using mixture of *Lactobacillus* sp. and *Bacillus* sp. growth and their antibacterial activity. Sci Rep 14:10224. 10.1038/s41598-024-59936-138702368 10.1038/s41598-024-59936-1PMC11068879

[17] Viswanathan S, Palaniyandi T, Shanmugam R, Karunakaran S, Pandi M, Wahab MRA, Baskar G, Rajendran BK, Sivaji A, Moovendhan M (2024) Synthesis, characterization, cytotoxicity, and antimicrobial studies of green synthesized silver nanoparticles using red seaweed *Champia parvula*. Biomass Conv Bioref 14:7387–7400. 10.1007/s13399-023-03775-z

[18] Nasr Azadani F, Madani M, Karimi J, Sepahvand S (2024) Green synthesis of silver nanoparticles by *Fusarium oxysporum* and its function against *Aspergillus* and *Fusarium* fungi. Indian J Microbiol 64(1):213–224. 10.1007/s12088-023-01162-w38468735 10.1007/s12088-023-01162-wPMC10924849

[19] Ullah H, Hefnawy M, Nawaz R, Baki ZA, Hanafiah MM, Anjum M (2025) Green synthesis of silver nanoparticles via ionic liquid mediated ultrasonic extracted secondary metabolites from *Amaranthus viridus* and their antibacterial performance. Chem Eng Commun. 10.1080/00986445.2025.2469590

[20] Venkatesham M, Ayodhya D, Madhusudhan A, Veera Babu N, Veerabhadram G (2014) A novel green one-step synthesis of silver nanoparticles using chitosan: catalytic activity and antimicrobial studies. Appl Nanosci 4:113–119. 10.1007/s13204-012-0180-y

[21] Zhang Y, Poon K, Masonsong GSP, Ramaswamy Y, Singh G (2023) Sustainable nanomaterials for biomedical applications. Pharmaceutics 15(3):922. 10.3390/pharmaceutics1503092236986783 10.3390/pharmaceutics15030922PMC10056188

[22] Aziz Mousavi SMA, Mirhosseini SA, Rastegar Shariat Panahi M, Mahmoodzadeh Hosseini H (2020) Characterization of biosynthesized silver nanoparticles using Lactobacillus rhamnosus GG and its in vitro assessment against colorectal cancer cells. Probiotics Antimicrob Proteins 12(2):740–74631020619 10.1007/s12602-019-09530-z

[23] Siddiqui AJ, Patel M, Jahan S, Abdelgadir A, Alam MJ, Alshahrani MM, Adnan M (2025) Silver nanoparticles derived from probiotic Lactobacillus casei—a novel approach for combating bacterial infections and cancer. Probiotics Antimicrob Proteins 17(3):1277–129438085438 10.1007/s12602-023-10201-3

[24] Das VL, Thomas R, Varghese RT, Soniya EV, Mathew J, Radhakrishnan EK (2014) Extracellular synthesis of silver nanoparticles by the *Bacillus strain* CS 11 isolated from industrialized area. 3 Biotech 4:121–126. 10.1007/s13205-013-0130-828324441 10.1007/s13205-013-0130-8PMC3964251

[25] Alfryyan N, Kordy MGM, Abdel-Gabbar M, Soliman HA, Shaban M (2022) Characterization of the biosynthesized intracellular and extracellular plasmonic silver nanoparticles using Bacillus cereus and their catalytic reduction of methylene blue. Sci Rep 12:12495. 10.1038/s41598-022-16029-135864132 10.1038/s41598-022-16029-1PMC9304349

[26] Prabhu S, Poulose EK (2012) Silver nanoparticles: mechanism of antimicrobial action, synthesis, medical applications, and toxicity effects. Int Nano Lett 2:32. 10.1186/2228-5326-2-32

[27] Vaidyanathan R, Gopalram S, Kalishwaralal K, Deepak V, Pandian SRK, Gurunathan S (2010) Enhanced silver nanoparticle synthesis by optimization of nitrate reductase activity. Colloids Surf B 75(1):335–341. 10.1016/j.colsurfb.2009.09.00610.1016/j.colsurfb.2009.09.00619796922

[28] Garg D, Sarkar A, Chand P, Bansal P, Gola D, Sharma S, Khantwal S, Surabhi, Mehrotra R, Chaucan N, Bharti RK (2020) Synthesis of silver nanoparticles utilizing various biological systems: mechanisms and applications—a review. Prog Biomater 9:81–95. 10.1007/s40204-020-00135-232654045 10.1007/s40204-020-00135-2PMC7544790

[29] Kabeerdass N, Otaibi A, Rajendran A, Manikandan M, Kashmery A, Rahman HA, Mathanmohun MM, M (2021) Bacillus-mediated silver nanoparticle synthesis and its antagonistic activity against bacterial and fungal pathogens. Antibiotics 10(11):133434827271 10.3390/antibiotics10111334PMC8614847

[30] Tariq F, Ahmed N, Afzal M, Khan MAU, Zeshan B (2020) Synthesis, characterization and antimicrobial activity of Bacillus subtilis-derived silver nanoparticles against multidrug-resistant bacteria. Jundishapur J Microbiol, 13(5)

[31] Afolayan EM, Afegbua SL, Ado SA (2023) Characterization and antibacterial activity of silver nanoparticles synthesized by soil-dwelling *Bacillus thuringiensi*s against drug-resistant bacteria. Biologia 78(8):2283–2292. 10.1007/s11756-023-01381-y

[32] Abeer Mohammed AB, Abd Elhamid MM, Khalil MKM, Ali AS, Abbas RN (2022) The potential activity of biosynthesized silver nanoparticles of Pseudomonas aeruginosa as an antibacterial agent against multidrug-resistant isolates from intensive care unit and anticancer agent. Environ Sci Eur 34(1):109

[33] Nadhe SB, Singh R, Wadhwani SA, Chopade BA (2019) Acinetobacter sp. mediated synthesis of AgNPs, its optimization, characterization, and synergistic antifungal activity against C. albicans. J Appl Microbiol 127(2):445–45831074075 10.1111/jam.14305

[34] Ekim B, Calik A, Ceylan A, Saçaklı P (2020) Effects of Paenibacillus xylanexedens on growth performance, intestinal histomorphology, intestinal microflora, and immune response in broiler chickens challenged with Escherichia coli K88. Poult Sci 99(1):214–22332416805 10.3382/ps/pez460PMC7587685

[35] Hwang J, Shin SC, Han JW, Hong SP, Min WK, Chung D, Kim HJ (2021) Complete genome sequence of Paenibacillus xylanexedens PAMC 22703, a xylan-degrading bacterium. Mar Genomics 55:10078832563695 10.1016/j.margen.2020.100788

[36] Zhang F, Li XL, Zhu SJ, Ojaghian MR, Zhang JZ (2018) Biocontrol potential of Paenibacillus polymyxa against verticillium dahliae infecting cotton plants. Biol Control 127:70–77

[37] Baindara P, Chaudhry V, Mittal G, Liao LM, Matos CO, Khatri N, Korpole S (2016) Characterization of the antimicrobial peptide penisin, a class Ia novel lantibiotic from Paenibacillus sp. strain A3. Antimicrob Agents Chemother 60(1):580–59126574006 10.1128/AAC.01813-15PMC4704198

[38] Amoah K, Dong XH, Tan BP, Zhang S, Chi SY, Yang QH, Zhang H (2021) Effects of three probiotic strains (*Bacillus coagulans*, *B. licheniformis* and *Paenibacillus polymyxa*) on growth, immune response, gut morphology and microbiota, and resistance against *Vibrio harveyi* of northern whitings, *Sillago sihama* Forsskål (1775). Anim Feed Sci Technol 277:114958

[39] Moon SG, Kothari D, Lee WD, Kim JI, Kim KI, Kim YG, Kim SK (2022) Potential probiotic acceptability of a novel strain of Paenibacillus Konkukensis SK 3146 and its dietary effects on growth performance, intestinal microbiota, and meat quality in broilers. Animals 12(11):147135681935 10.3390/ani12111471PMC9179277

[40] Alagawany M, Madkour M, El-Saadony MT, Reda FM (2021) *Paenibacillus polymyxa* (LM31) as a new feed additive: antioxidant and antimicrobial activity and its effects on growth, blood biochemistry, and intestinal bacterial populations of growing Japanese quail. Anim Feed Sci Technol 276:114920

[41] Calik A, Burcu EKİM, Bayraktaroğlu AG, Ergün A, Saçaklı P (2017) Effects of dietary probiotic and synbiotic supplementation on broiler growth performance and intestinal histomorphology. Ankara Üniversitesi Veteriner Fakültesi Dergisi 64(3):183–189

[42] Ekim B, Doğan SY, Özdemir C (2017) Spread of infection to poultry challenged with *E.coli* K88 and fed different diets. Bulg J Vet Med 20(1):333–337

[43] Qiu M, Tian Y, Qu W, Ma Y, Zhao F, Jiang Y, Man C (2025) Postbiotic-biosynthesized silver nanoparticles anchored on covalent organic frameworks integrated into carboxymethyl chitosan-based film for enhancing antibacterial packaging. Int J Biol Macromol 291:13914339722393 10.1016/j.ijbiomac.2024.139143

[44] Ranjani S, Hemalatha S (2025) Probiotic bacteria Bacillus licheniformis mediated sustainable green synthesis of nanoparticles and its multifaceted mechanisms to control dermal pathogens. Mater Chem Phys, 131261

[45] Doğan SY (2024) Antimicrobial properties of lyophilized Lemna minor L. extract obtained by microwave extraction. *International Journal of Science, Engineering and Management*, *11*(5)

[46] Clinical and Laboratory Standards Institute (CLSI) (2023) Performance Standards for Antimicrobial Susceptibility Testing; 33rd ed. CLSI supplement M100

[47] Doğan SY (2025) Cytotoxic and apoptotic effect of *lemna minor* L. extract on human osteosarcoma (Saos-2). Int J Second Metab 12(2):321–330

[48] Kaya S, Solak EK, Doğan SY, Demirkaya A, Celep AGS (2024) Designing of polymeric nanoparticles for enhanced breast cancer therapy: combining Paclitaxel, boric acid and Tannic acid for controlled drug delivery. ChemistrySelect, 9(7), e202304672

[49] Shaban M, Kholidy I, Ahmed GM, Negem M, El-Salamb HMA (2019) Cyclic voltammetry growth and characterization of Sn–Ag alloys of diferent nanomorphologies and compositions for efcient hydrogen evolution in alkaline solutions. R Soc Chem 9:22389–22400 10.1039/c9ra03503f PMC9066623 35519441

[50] Wagi S, Ahmed A (2019) Green production of AgNPs and their phytostimulatory impact. De Gruyter 8:885–894

[51] Hui Y, Yan-yu R, Tao W, Chuang W (2016) Preparation and antibacterial activities of Ag/Ag+/Ag3 + nanoparticle composites made by pomegranate (*Punica granatum*) rind extrac. Res Phys 6:299–304

[52] Ali J, Ali N, Jamil SUU, Waseem H, Khan K, Pan G (2017) Insight into eco-friendly fabrication of silver nanoparticles by *Pseudomonas aeruginosa* and its potential impacts. J Environ Chem Eng 5(4):3266–3272. 10.1016/j.jece.2017.06.038

[53] Singh P, Pandit S, Mokkapati V, Garnæs J, Mijakovic IA (2020) Sustainable approach for the green synthesis of silver nanoparticles from *Solibacillus isronensis* sp. and their application in biofilm inhibition. Molecules 25:2783. 10.3390/molecules2512278332560208 10.3390/molecules25122783PMC7355478

[54] Balaji DS, Basavaraja S, Deshpande R, Mahesh DB, Prabhakar BK, Venkataraman A (2009) Colloids Surf B Biointerfaces 68(1):88–9218995994 10.1016/j.colsurfb.2008.09.022

[55] Elumalai D, Ashok K, Suresh A, Hemavathi M (2016) Green synthesis of silver nanoparticle using *Achyranthes aspera* and its larvicidal activity against three major mosquito vectors. Eng Agric Environ Food 9(1):1–8

[56] Okumus E (2024) Green synthesis of silver nanoparticles using *Hebeloma excedens* mushroom extract as a new source: anti-lipid peroxidation, bioaccessibility and antidiabetic properties. J Food Meas Charact 18(6):5157–5169

[57] Chifiriuc MC, Filip R, Constantin M, Pircalabioru GG, Bleotu C, Burlibasa L, Mihaescu G (2022) Common themes in antimicrobial and anticancer drug resistance. Front Microbiol 13:96069336003940 10.3389/fmicb.2022.960693PMC9393787

[58] Lamaudière MT, Arasaradnam R, Weedall GD, Morozov IY (2023) The colorectal cancer gut environment regulates activity of the microbiome and promotes the multidrug resistant phenotype of ESKAPE and other pathogens. mSphere 8(2):e00626-2236847529 10.1128/msphere.00626-22PMC10117110

[59] Naz SS, Ortega S, J (2025) The role of Next-Generation probiotics in colorectal cancer pathways: mechanisms and therapeutic potential. Probiotics and Antimicrobial Proteins, pp 1–21 10.1007/s12602-025-10745-6 40889060

[60] Nguyen NPU, Dang NT, Doan L, Nguyen TTH (2023) Synthesis of silver nanoparticles: from conventional to ‘modern’methods—a review. Processes 11(9):2617

[61] Huq MA (2020) Paenibacillus Anseongense sp. nov. A silver nanoparticle producing bacterium isolated from rhizospheric soil. Curr Microbiol, 77(9) 10.1007/s00284-020-02086-0 32556479

[62] Sreenivasa N, Meghashyama BP, Pallavi SS, Bidhayak C, Dattatraya A, Muthuraj R, Vaishnavi MD (2021) Biogenic synthesis of silver nanoparticles using *Paenibacillus* sp. in-vitro and their antibacterial, anticancer activity assessment against human colon tumour cell line. J Environ Biol 42(1):118–127

[63] El Shanshoury AERR, Sabae SZ, Shouny E, Elsaied WA, Badr HE, Abo-Shady HM, A. M (2023) Biomimetic synthesis of silver nanoparticles using new aquatic species of Bacillus, Alcaligenes, and Paenibacillus and their potential antibiofilm activity against Biofilm-Forming Escherichia coli. Lett Appl NanoBioSci, 12

[64] Huq MA, Khan AA, Alshehri JM, Rahman MS, Balusamy SR, Akter S (2023) Bacterial mediated green synthesis of silver nanoparticles and their antibacterial and antifungal activities against drug-resistant pathogens. Royal Society Open Science 10(10):23079637885988 10.1098/rsos.230796PMC10598446

[65] Tregubova KV, Yegorenkova IV, Grinev VS, Fomin AS (2023) Biological activity of silver nanoparticles synthesized with *Paenibacillus polymyxa* exopolysaccharides. Enzyme Microb Technol 164:11017436508942 10.1016/j.enzmictec.2022.110174

[66] Fahim M, Shahzaib A, Nishat N, Jahan A, Bhat TA, Inam A (2024) Green synthesis of silver nanoparticles: a comprehensive review of methods, influencing factors, and applications. JCIS Open 16:100125

[67] Di Fraia A, Dal Poggetto G, Salamone M, Carraturo F, Contursi P, Guida M, Fiorentino G (2025) Green synthesis of silver nanoparticles (AgNPs) from *G. stearothermophilus* GF16: stable and versatile nanomaterials with antioxidant, antimicrobial, and catalytic properties. Microb Cell Fact 24(1):18940830875 10.1186/s12934-025-02815-9PMC12362984

[68] El-Hawary AS, Ibrahim OM, Kalaba MH, El-Sehrawy MH, Ismail MK (2025) *Limosilactobacillus fermentum*-derived silver nanoparticles: biosynthesis, optimization, and biological activities. Biomass Convers Biorefin 15(7):9999–10013

[69] Jiang XC, Chen WM, Chen CY, Xiong SX, Yu AB (2010) Role of temperature in the growth of silver nanoparticles through a synergetic reduction approach. Nanoscale Res Lett 6(1):3227502655 10.1007/s11671-010-9780-1PMC3211407

[70] Htwe YZN, Chow WS, Suda Y, Mariatti M (2019) Effect of silver nitrate concentration on the production of silver nanoparticles by green method. Materials Today: Proceedings 17:568–573

[71] Paul TK, Jalil MA, Repon MR, Alim MA, Islam T, Rahman ST, Rhaman M (2023) Mapping the progress in surface plasmon resonance analysis of phytogenic silver nanoparticles with colorimetric sensing applications. Chem Biodivers, 20(8), e202300510 10.1002/cbdv.202300510 37471642

[72] John MS et al (2020) Synthesis of bioactive silver nanoparticles by a Pseudomonas strain associated with the Antarctic psychrophilic protozoon euplotes focardii. Mar Drugs 18(1):1–13 10.3390/md18010038 PMC7024347 31947807

[73] Awwad AM, Salem NM, Abdeen AO (2013) Green synthesis of silver nanoparticles using carob leaf extract and its antibacterial activity. Int J Ind Chem 4(29):1–6

[74] Firdhouse M, Lalitha J (2015) Biosynthesis of silver nanoparticles and its applications. J Nanotechnol 2015:1–18

[75] Castañeda-Aude JE, Morones-Ramírez JR, De Haro-Del Río DA, León-Buitimea A, Barriga-Castro ED, Escárcega-González CE (2023) Antibiotics 12(3):57436978442 10.3390/antibiotics12030574PMC10044608

[76] Duman H, Eker F, Akdaşçi E, Witkowska AM, Bechelany M, Karav S (2024) Silver nanoparticles: a comprehensive review of synthesis methods and chemical and physical properties. Nanomaterials 14(18):152739330683 10.3390/nano14181527PMC11434896

[77] Abbai R, Govender P, Kannan P (2022) Strong antimicrobial activity of silver nanoparticles obtained by microbial supernatant: FTIR results indicate the presence of carboxyl and amine groups as stabilizing biomolecules. BMC Nanosci 12:64

[78] Pasieczna-Patkowska S, Michalczyk-Mazurek M, Kozak M, Giersig M (2023) Application of Fourier Transform Infrared (FTIR) spectroscopy in characterization of green-synthesized nanoparticles: a systematic review. Molecules 30(3):684. 10.3390/molecules3003068410.3390/molecules30030684PMC1182121039942788

[79] Magro L, Maragno AM, Vianello A, Della Ventura B (2021) Influence of the capping of biogenic silver nanoparticles on their physico-chemical and biological activity. J Nanobiotechnol 19:97. 10.1186/s12951-021-00797-5

[80] Abishad P, Vergis J, Unni V, Ram VP, Niveditha P, Yasur J, Juliet S, John L, Byrappa K, Nambiar P, Kurkure NV, Barbuddhe SB, Rawool DB (2022) Green synthesized silver nanoparticles using *Lactobacillus acidophilus* as an antioxidant, antimicrobial, and antibiofilm agent against multi-drug resistant enteroaggregative *Escherichia coli*. Probiotics Antimicrob Proteins 14:904–914. 10.1007/s12602-022-09961-135715714 10.1007/s12602-022-09961-1

[81] Lateef A, Ojo SA, Akinwale AS, Azeez L, Gueguim-Kana EB, Beukes LS (2015) Biogenic synthesis of silver nanoparticles using cell-free extract of *Bacillus safensis* LAU 13: antimicrobial, free radical scavenging and larvicidal activities. Biologia 70(10):1295–1306

[82] Ibrahim S, Ahmad Z, Manzoor MZ, Mujahid M, Faheem Z, Adnan A (2021) Optimization for biogenic microbial synthesis of silver nanoparticles through response surface methodology, characterization, their antimicrobial, antioxidant, and catalytic potential. Sci Rep 11(1):77033436966 10.1038/s41598-020-80805-0PMC7804320

[83] Jeyachandran S, Giri J, Alarifi A (2025) Biogenic synthesis of *Bacillus lentus*-mediated silver nanoparticles and its multifaceted applications in antibacterial, anti-biofilm, anti-larvicidal and anticancer activities. Green Chem Lett Rev 18(1):2503730

[84] Liu HY, Prentice EL, Webber MA (2024) Mechanisms of antimicrobial resistance in biofilms. NPJ Antimicrob Resist 2(1):2739364333 10.1038/s44259-024-00046-3PMC11445061

[85] Nazeer RR, Askenasy I, Swain JEV et al (2024) Contribution of the infection ecosystem and biogeography to antibiotic failure in vivo. NPJ Antimicrob Resist 2:45. 10.1038/s44259-024-00063-239649078 10.1038/s44259-024-00063-2PMC11618093

[86] Alghurabi MN, Mubarak TH, Judran AK, Hasoon BA (2025) Two-stage pulsed laser ablation for the production of Ag@ TiO2 core–shell nanoparticles with enhanced antimicrobial properties: an in silico study. Plasmonics. 10.1007/s11468-025-03143-9

[87] Sherry L, Rajendran R, Lappin DF, Borghi E, Fallahi S, Nile CJ (2014) Biofilm formation of *Candida albicans* and its impact on antifungal susceptibility. Crit Rev Microbiol 40(4):329–345. 10.3109/1040841X.2013.87903323215777

[88] Jawad KH, Hasen TS, Hammoud D, Hasoon BA, Jabir MS, Ghotekar S, Swelum AA (2025) Eco-friendly methods for synthesis of MgS@ CuS nanocomposite for overcoming multidrug resistance bacteria: insilico assessment study. J Drug Deliv Sci Technol, 106998

[89] Najm MAA, Shakir HA, Hasen ST et al (2025) Antimicrobial mechanisms and mode of actions of nanoemulsion against drug-resistant ESKAPE pathogens. Sci Rep 15:18014. 10.1038/s41598-024-02543-740410449

[90] Nguyen KN, Sao L, Kyllo K, Hernandez D, Salomon S, Shah K, Kao KC (2024) Antibiofilm activity of PDMS/TiO2 against *Candida glabrata* through inhibited hydrophobic recovery. ACS Omega 9(41):42593–4260139431067 10.1021/acsomega.4c07869PMC11483912

[91] Ramalingam K, Khan MH (2022) Antimicrobial mechanisms and mode of actions of nanoemulsion against drug-resistant ESKAPE pathogens. Handbook of research on nanoemulsion applications in Agriculture, Food, Health, and biomedical sciences. IGI Global Scientific Publishing, pp 142–168

[92] Sami RH, Jawad SF, Zankanah FH, Jawad KH, Hasoon BA, Issa AA, Al-azawi KF (2025) Biosynthesis of platinum nanoparticles: evaluation of their activity against *streptococcus mutans* and in silico study. Plasmonics 20(6):3413–3427

[93] Mostafavi E, Zarepour A, Barabadi H, Zarrabi A, Truong LB, Medina-Cruz D (2022) Antineoplastic activity of biogenic silver and gold nanoparticles to combat leukemia: beginning a new era in cancer theragnostic. Biotechnol Rep, 34, e00714 10.1016/j.btre.2022.e00714 PMC9171450 35686001

[94] Almukaynizi FB, Daghestani MH, Awad MA, Althomali A, Merghani NM, Bukhari WI, Bhat RS (2022) Cytotoxicity of green-synthesized silver nanoparticles by *Adansonia digitata* fruit extract against HTC116 and SW480 human colon cancer cell lines. Green Process Synth 11(1):411–422

[95] Alobaid HM, Daghestani MH, AL-Malahi NM, Alzahrani SA, Hassen LM, Metwally DM (2022) Exploring the effect of silver nanoparticles on gene expression in colon cancer cell line HCT116. Green Process Synth 11(1):1108–1117

[96] Gurunathan S, Qasim M, Park C, Yoo H, Kim JH, Hong K (2018) Cytotoxic potential and molecular pathway analysis of silver nanoparticles in human colon cancer cells HCT116. Int J Mol Sci 19(8):226930072642 10.3390/ijms19082269PMC6121495

[97] Zhai Y, Wang B, Han W, Yu B, Ci J, An F (2023) Green synthesis of AgNPs using plant extract and investigation of its anti-human colorectal cancer application. Open Chem 21(1):20230174

[98] Susanto H, Firdaus SDRA, Sholeh M, Endharti AT, Taufiq A, Malek NANN, Permatasari HK (2024) *Moringa oleifera* leaf powder–silver nanoparticles (MOLP-AgNPs) efficiently inhibit metastasis and proliferative signaling in HT-29 human colorectal cancer cells. J Agric Food Res 16:101149

[99] Khajeh H, Fazeli-Nasab B, Pourshahdad A, Mirzaei AR, Ghorbanpour M (2024) Green-synthesized silver nanoparticles induced apoptotic cell death in CACO2 cancer cells by activating MLH1 gene expression. Sci Rep 14(1):2960139609574 10.1038/s41598-024-80809-0PMC11604737

[100] The International Organization for Standardization (ISO) (2009) Biological evaluation of medical devicesPart 5: Tests for in vitro cytotoxicity (Edition 3); ISO 10993-5:2009

